# Predicting associations among drugs, targets and diseases by tensor decomposition for drug repositioning

**DOI:** 10.1186/s12859-019-3283-6

**Published:** 2019-12-16

**Authors:** Ran Wang, Shuai Li, Lixin Cheng, Man Hon Wong, Kwong Sak Leung

**Affiliations:** 1 Department of Computer Science and Engineering, The Chinese University of Hong Kong, Hong Kong, China; 2 Department of Critical Care Medicine, Shenzhen People’s Hospital, The Second Clinical Medicine College of Ji’nan University, Shenzhen, China

**Keywords:** Drug repositioning, Drug-target-disease associations, Tensor decomposition, Clustering

## Abstract

**Background:**

Development of new drugs is a time-consuming and costly process, and the cost is still increasing in recent years. However, the number of drugs approved by FDA every year per dollar spent on development is declining. Drug repositioning, which aims to find new use of existing drugs, attracts attention of pharmaceutical researchers due to its high efficiency. A variety of computational methods for drug repositioning have been proposed based on machine learning approaches, network-based approaches, matrix decomposition approaches, etc.

**Results:**

We propose a novel computational method for drug repositioning. We construct and decompose three-dimensional tensors, which consist of the associations among drugs, targets and diseases, to derive latent factors reflecting the functional patterns of the three kinds of entities. The proposed method outperforms several baseline methods in recovering missing associations. Most of the top predictions are validated by literature search and computational docking. Latent factors are used to cluster the drugs, targets and diseases into functional groups. Topological Data Analysis (TDA) is applied to investigate the properties of the clusters. We find that the latent factors are able to capture the functional patterns and underlying molecular mechanisms of drugs, targets and diseases. In addition, we focus on repurposing drugs for cancer and discover not only new therapeutic use but also adverse effects of the drugs. In the in-depth study of associations among the clusters of drugs, targets and cancer subtypes, we find there exist strong associations between particular clusters.

**Conclusions:**

The proposed method is able to recover missing associations, discover new predictions and uncover functional clusters of drugs, targets and diseases. The clustering of drugs, targets and diseases, as well as the associations among the clusters, provides a new guiding framework for drug repositioning.

## Background

Developing a new drug experimentally is a time-consuming and costly process. The process usually takes 13-15 years from the start of developing a new drug to getting it into market and costs 2-3 billion US dollars on average by report. However, the costs are still increasing these years while the number of drugs approved by the US Food and Drug Administration (FDA) every year per dollar spent on development is declining [1]. The gap between the therapeutic needs and the limited number of available drugs is a big challenge to pharmaceutical research [2]. With the advances in genomics, proteomics and systems biology, large amounts of omics data are accumulated and promote the development of computational methods for drug discovery. Drug repositioning, one of the approaches of drug discovery, aims to find new therapeutic use of existing drugs that have passed a significant number of toxicity and other tests and have been approved by the regulatory agencies. Some estimates suggest that repositioning a drug takes around 6.5 years and costs $300 million on average, which is more efficient and much cheaper than the traditional drug development process, and attracts a lot attention of pharmaceutical researchers.

A variety of computational methods for drug repositioning have been proposed. The basic strategy is to either find new targets for existing drugs or discover new drug-disease associations. The fundamental assumption is that agents with similar properties have similar therapeutic effects. The proposed methods of drug repositioning can be categorized into three groups: machine learning-based methods, matrix decomposition-based methods and network-based methods. In machine learning-based methods, each drug, target and disease is represented by a feature vector based on their properties, such as chemical structures, side effects and fingerprints of drugs, genomic characters of targets, and phenotype information of diseases. Then the machine learning models is trained on the feature vectors and further provides new predictions of associations [3, 4]. On account of the strong predictive power of deep learning methods in recent years, various deep learning models are applied to drug repositioning, including multi-layer perceptron [5, 6], deep belief network [7] stacked auto-encoder [8, 9], etc. When training the machine learning models, both positive and negative training samples should be provided. However, it is hard to select negative samples since there are rarely experimentally verified negative samples in this field.

Drug repositioning is analogous to recommendation systems, since it aims to recommend potential drugs for diseases. Therefore, matrix decomposition methods, which are widely used in recommendation systems, are applied to drug repositioning. Different kinds of information are used to measure the similarity of drugs, targets and diseases, including chemical structures, genetic variations and gene expression profiles. Xuan et al. [10] measured the similarity of disease based on their semantic similarity and Disease Ontology (DO). Zheng et al. [11] proposed a collaborative matrix factorization method combining more than one similarity matrices of drugs and targets. They demonstrated that the same calculation of similarity performed differently on different datasets, while different calculations of similarity also performed differently on the same dataset. Cobanoglu et al. [12] performed probabilistic matrix factorization (PMF) on drug repositioning and analyzed drug clusters derived from PMF latent factors. Luo et al. [13] constructed a heterogeneous network by integrating drug-drug, disease-disease and drug-disease networks, and adopted Singular Value Thresholding (SVT) algorithm on the adjacency matrix.

In order to integrate heterogeneous data, network-based methods are applied to drug repositioning. Diverse properties of drugs, targets and diseases have been used to construct heterogeneous networks [14–16]. Luo et al. [17] extracted low dimensional but informative vector representations of drugs and proteins from different networks, which were further used to find the best projection from drug space to protein space. Lee et al. [18] integrated protein-protein interactions (PPIs) and gene regulations to their network. Semantic meanings of different meta-paths in the heterogeneous network were considered in [19].

Another strategy used for drug repositioning is profile-based, using signature reversion techniques to find drug-disease pairs that have anti-correlated expression profiles. Kim et al. [20] identified drug candidates for gastric cancer using a computational reversal of gene expression approach. Nagaraj et al. [21] found disease-associated gene enriched mutational phenotype profiles of mouse, and then prioritized drugs based on the similarities between phenotype profiles of diseases and drugs. In order to remove effects of background of tissues, Xu et al. [22] derived non-tissue-specific core signatures (CSs) and identified drugs with perturbation signatures. The profile-based methods do not require prior knowledge on associations of diseases or drugs. However, noisy profiles might lead to higher false positives when a drug or disease does not demonstrate a strong perturbation on gene expression. Moreover, the methods might also fail when the observed alterations are consequences of the disease but not causes [2].

Recently, some other molecules are found acting as therapeutic targets of drugs, such as miRNAs, which are used to repurpose drugs for corresponding diseases [23]. There are also studies investigating drug repositioning from other perspectives. For example, Iwata et al. [24] regarded molecular pathways as therapeutic targets and discovered drug-disease associations by identifying drugs that could inactivate the cancer-growth involved pathways or activate cancer-death related pathways of the disease. The studies mentioned above investigate pairwise associations among drugs, proteins, genes, diseases, pathways, etc. However, most of the FDA-approved drugs were developed with the underlying molecular mechanisms uncovered [14]. Thus, it is of significant importance to discover the whole picture of drug-target-disease associations to understand the underlying mechanisms.

In this paper, we propose a novel framework for drug repositioning investigating drug-target-disease (DTD) triplet associations. First, we construct three-dimensional tensors representing DTD associations and decompose the tensors to derive latent factors and discover new predictions. We investigate the role of different additional information related to drugs and targets and the effects of other factors. Then we examine the ability of predicting new associations of the proposed method. The latent factors derived from the association tensor are analyzed to uncover functional patterns of drugs, targets and diseases. Finally, we apply the proposed method to a cancer data set and identify drug candidates for several cancers.

## Results

### Recovering missing associations

**Fig. 1:**
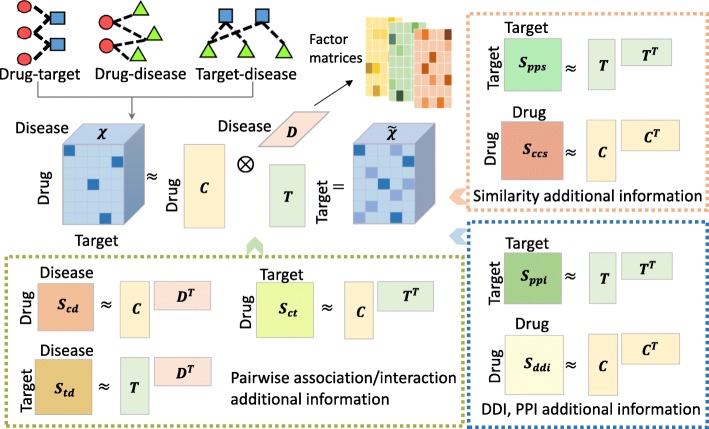
Workflow of the proposed method. The association tensor is integrated from the pairwise associations, including drug-target interactions, drug-disease associations and target-disease associations. It is decomposed, together with additional information, into three factor matrices

Figure 1 illustrates the workflow of the proposed method. We construct two association tensors, *χ*^*tri*^ and *χ*^*bi*^, based on the DTD subset using different strategies (Methods). Each entry in *χ*^*tri*^ represents the existence of the corresponding triplet association, meaning that for the corresponding drug, target and disease, all of the three pairwise associations exist. Each triplet association in *χ*^*bi*^ indicates that for the corresponding drug, target and disease, the drug is associated with the target and the target is associated with the disease, so that it is inferred that the drug is associated with the disease. Each association tensor is decomposed together with different kinds of additional information separately, resulting in three factor matrices for drugs, targets and diseases, respectively. We test the ability of recovering missing associations of the proposed method by 10-fold cross-validation, and use area under the receiver operating characteristic curve (AUC) as well as area under precision-recall curve (AUPR) to evaluate the performance (Methods).

**Fig. 2:**
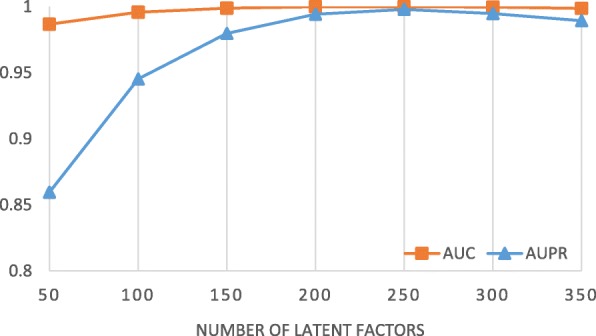
Performance of decomposing *χ*^*bi*^ with different number of latent factors. Similarity of drugs and targets is used as additional information

We find that the AUC and AUPR increase as the number of latent factors increases until the number of latent factors approaches 250 (Fig. 2). One possible reason is that more latent factors have higher ability to characterize the latent patterns of associations, so that they can approximate the tensor better. However, when the number of latent factors increases further, the performance drops because the derived latent factors are over-fitted to the observations in the association tensor and hence have lower generalization ability.

### Factors affecting the performance

We investigate the factors that might affect the performance of recovering missing associations, including the tensor construction strategy, the use of different kinds of additional information, and the sparseness of the tensor.

**Fig. 3:**
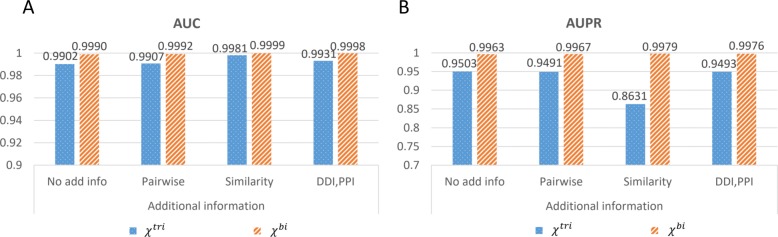
Performance of decomposing association tensors constructed from different strategy. **a** Comparison of AUC. **b** Comparison of AUPR. No add info, using no additional information. Pairwise, using pairwise associations as additional information. Similarity, using similarity of drugs and targets as additional information. DDI, PPI, using DDIs and PPIs as additional information. The number of latent factors is set to 250

We compare the performance of decomposing *χ*^*tri*^ and *χ*^*bi*^, which are constructed from different strategies. The AUC of decomposing *χ*^*tri*^ and *χ*^*bi*^ are comparable (Fig. 3a). However, the decomposition of *χ*^*bi*^ outperforms that of *χ*^*tri*^ in AUPR (Fig. 3b), indicating that the reconstructed $\widetilde {\chi }^{tri}$ (compared to *χ*^*tri*^) gives much more false positives than the reconstructed $\widetilde {\chi }^{bi}$ (compared to *χ*^*bi*^). It is possible that some of the false positives in *χ*^*tri*^ decomposition might be true positives in *χ*^*bi*^ decomposition, since there are more observations in *χ*^*bi*^ compared to *χ*^*tri*^.

**Fig. 4:**
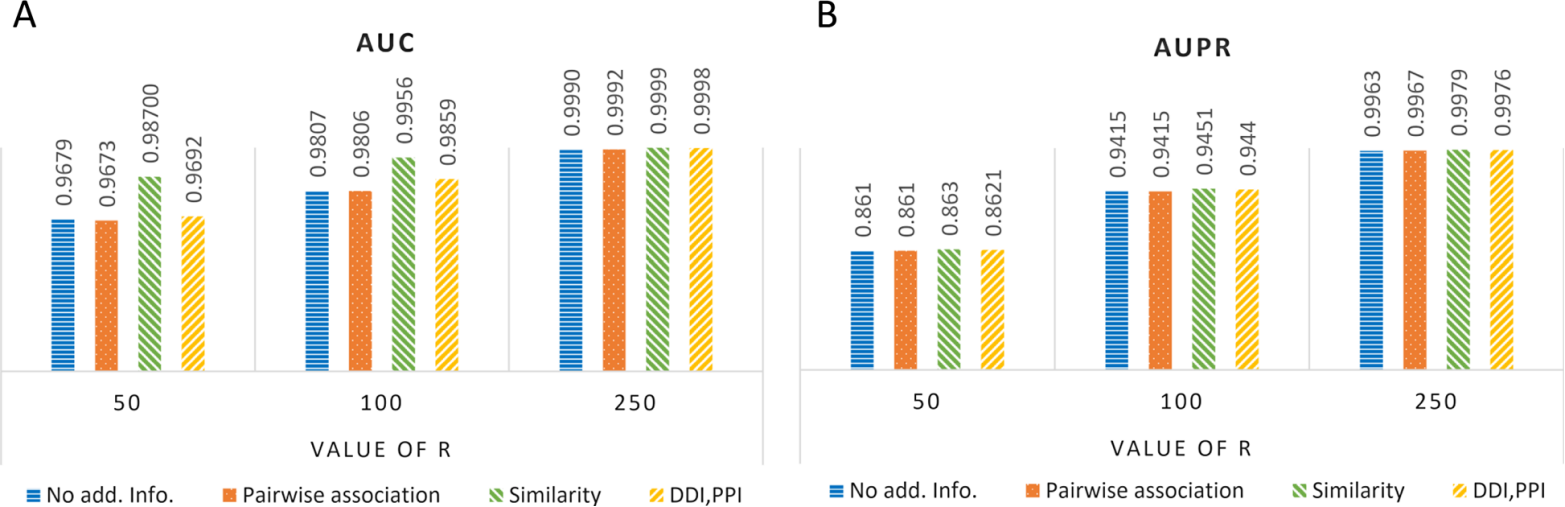
Performance of decomposing *χ*^*bi*^ with different additional information. AUC (**a**) and AUPR (**b**) evaluated under different number of latent factors (R) are illustrated. No add. info., using no additional information

Figure 4 demonstrates the performance of decomposing *χ*^*bi*^ with different additional information, including similarities of drugs and targets, pairwise associations, as well as drug-drug interactions (DDIs) and PPIs (Methods). When the number of latent factors is small, information of similarity helps a lot in improving the performance, which is consistent with the fundamental assumption in drug repositioning that similar drugs and targets have similar functional effects. As the number of latent factors increases, the advantage of using additional information becomes smaller since the large factor matrices are able to characterize the patterns of the triplet associations. However, when decomposing *χ*^*tri*^, information of similarity induces worse performance in AUPR (Additional file 1: Figure S1). One possible reason is that some of the triplet associations are recovered according to the information of similarity, but these associations do not exist in *χ*^*tri*^, so that they are regarded as false positives, which might exist in *χ*^*bi*^.

**Fig. 5:**
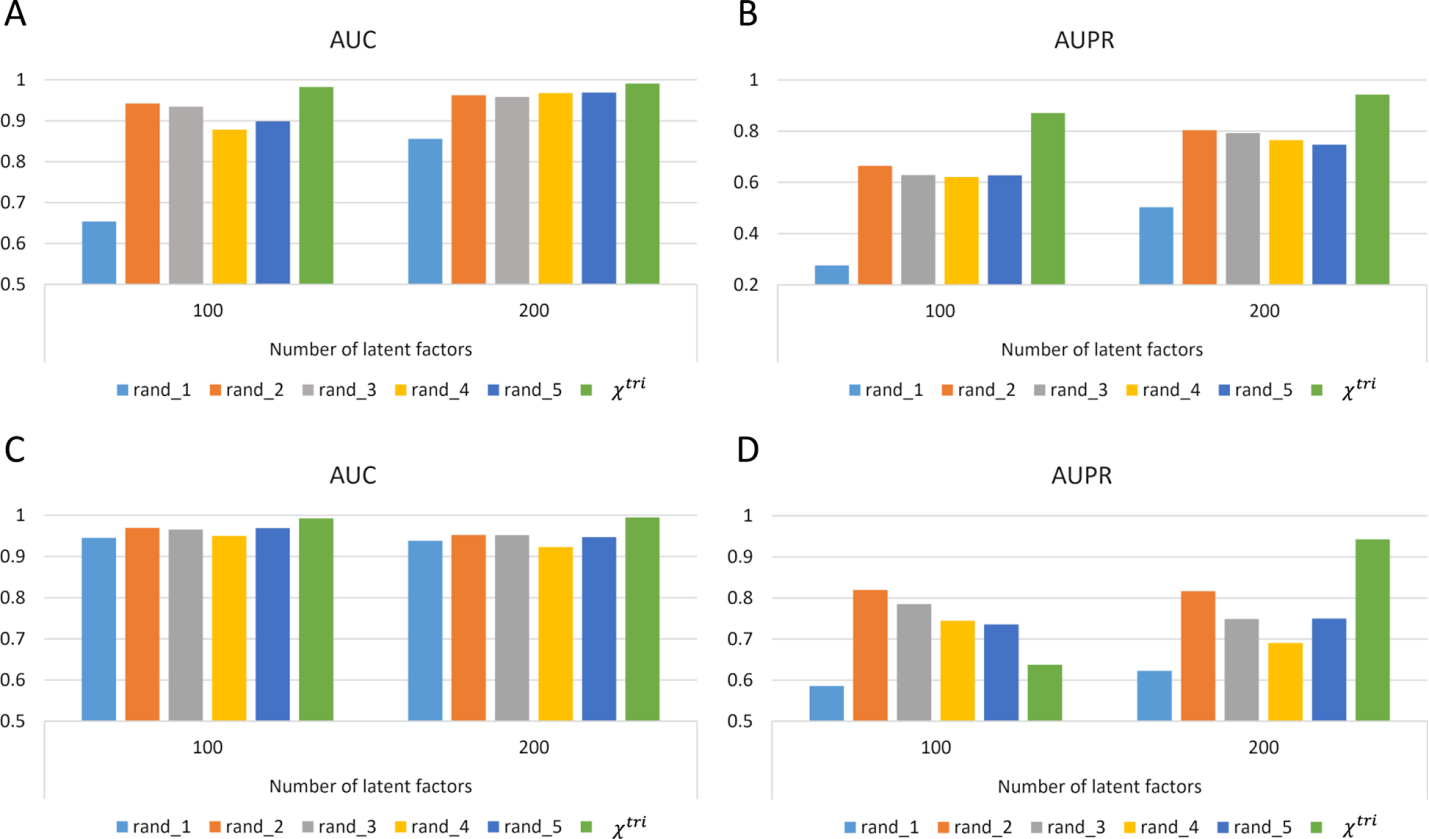
Performance of decomposing the five random tensors constructed by the first strategy. Performance of using different number of latent factors is demonstrated, compared with performance of decomposing *χ*^*tri*^. **a** and **b**, AUC and AUPR with no additional information, respectively. **c** and **d**, AUC and AUPR using similarity as additional information, respectively

Since the association tensors are very sparse and sparsity is always a crucial problem in recommendation systems, it is of interest to investigate the effects of sparseness on the performance of tensor decomposition. Therefore, we generate five pairs of tensors with different sparseness from the original data set [17] instead of DTD subset. The two tensors in each pair, analogous to *χ*^*tri*^ and *χ*^*bi*^, are constructed from the same set of drugs, targets and diseases but using different strategies, respectively, while the drugs, targets and diseases used in different pairs are selected randomly (Methods). The proportions of observed associations in the five pairs are summarized in Table 1. Figure 5 illustrates the performance of decomposing the random tensors constructed by the first strategy without any additional formation (Fig.5a, 5b) and using similarity as additional information (Fig.5c, 5d). The performance of decomposing random tensors constructed by the second strategy is illustrated in (Additional file2: Figure S2). Unexpectedly, the second and third random tensor pairs, which have the highest sparseness, however, perform better than or comparable to the other pairs. On the contrary, the first random tensor pair with relatively low sparseness performs the worst. The results indicate that the sparseness of association tensors is not determinant to the performance.

**Table 1:** Statistics of proportion of observed data in the random tensors

Pairs	rand1	rand2	rand3	rand4	rand5
Strategy 1	3.12e-5	1.16e-6	8.67e-7	6.88e-5	7.97e-5
Strategy 2	1.96e-4	5.99e-6	4.76e-6	4.09e-4	4.21e-4

**Fig. 6:**
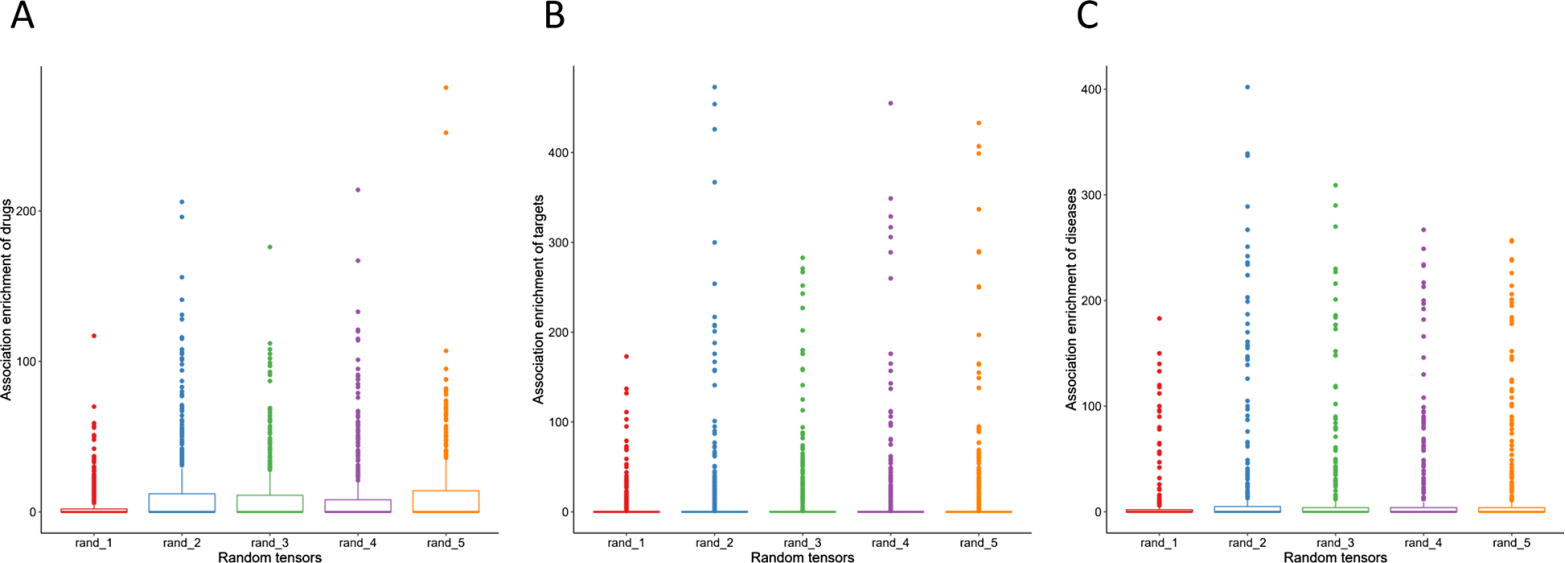
Boxplot of association enrichment. Association enrichment of drugs (**a**), targets (**b**) and diseases (**c**) in the five random tensors constructed by the first strategy are illustrated

**Fig. 7:**
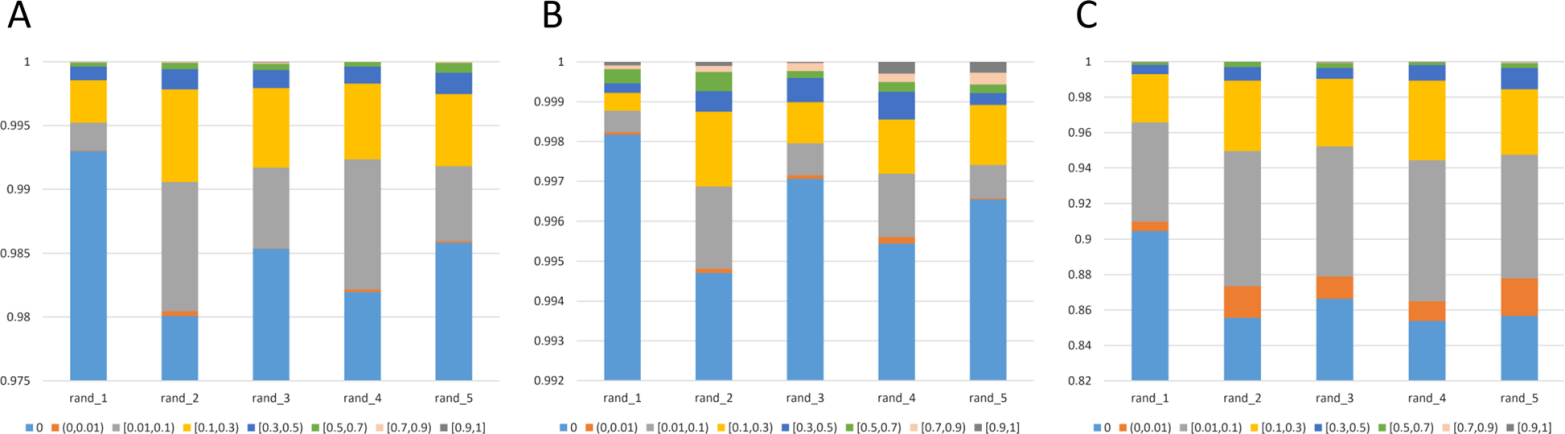
Similarity of triplet associaiton patterns of drug pairs (**a**), target pairs (**b**) and disease pairs (**c**) in the five random tensors constructed by the first strategy. X-axis represents different random tensors. Y-axis represents the cumulative proportion of different ranges of Jaccard similarity. Different colors demonstrate different ranges of similarity

In order to find other factors respect to data quality that might affect the performance instead of sparseness, we further look into details of the triplet associations in the random tensor pairs, including the association enrichment of individual drugs, targets and diseases, as well as the similarity of their association patterns (Methods). Association enrichment of a drug/target/diseases is defined as the total number of corresponding triplet associations, measuring the amount of functional information provided for each drug/target/disease in the tensor. We find that in the first random tensor pair, which performs the worst among all the pairs, the association enrichment of the drugs, targets and diseases are lower compared to the other pairs (Fig. 6, Additional file 3: Figure S3). It means that the associations are distributed more equally to all the drugs, targets and diseases but fewer to each in the first pair, while distributed more unbalanced to all but more to part of them in the other pairs. More unbalanced associations might result in factor matrices which better characterize some of the drugs, targets and diseases, and further contribute to recovering unknown associations. In addition, we investigate the pairwise-similarity of drugs, targets and diseases in terms of their association patterns in each random tensor pair, which is calculated by the Jaccard similarity of vector-represented triplet associations (Methods). The drugs, targets and diseases in the first random tensor pair demonstrate lower pairwise-similarity in terms of association patterns (Fig. 7, Additional files 4, 5, 6: Figure S4-S6), which might has an impact on the performance. The pairwise-similarity in terms of associations patterns of drugs, targets and diseases in *χ*^*tri*^ and *χ*^*bi*^ are much higher compared to the random tensors (Additional file 7, 8: Figure S7-S8), which might be a reason for the better performance of decomposing *χ*^*tri*^ and *χ*^*bi*^. Taking all above into consideration, it implies that the proposed method makes use of existing triplet associations of drugs, targets and diseases as well as similar behavioral patterns to infer missing associations. Association enrichment and similar functional drugs, targets and diseases are more important to the performance than sparseness. Fortunately, comparing the performance of using no additional information (Fig. 5a, 5b) and using similarity as additional information (Fig. 5c, 5d), the use of additional information helps a lot when the quality of associations tensor is poor.

### Comparison with Baseline methods

We compare the proposed method with three baseline methods, which are SNScore [25], Network-based Random Walk with Restart on Heterogeneous network (NRWRH) [14] and Collective Matrix Factorization (CMF) (Methods, Additional file 9: Figure S9), using 10fold cross-validation. However, all of the three baselines aim to infer pairwise associations among drugs, targets and diseases instead of triplet associations. In order to evaluate their capability of inferring triplet associations and compare to the proposed method, we first project the randomly selected triplet associations (test samples) in cross-validation to pairwise associations, randomly mask one of the three pairwise associations of each test sample, and predict the masked pairwise associations using the baseline methods. Then, the inferred score of the test samples is calculated by the product of the inferred probability or known binary value of the corresponding pairwise associations (Methods).

**Fig. 8:**
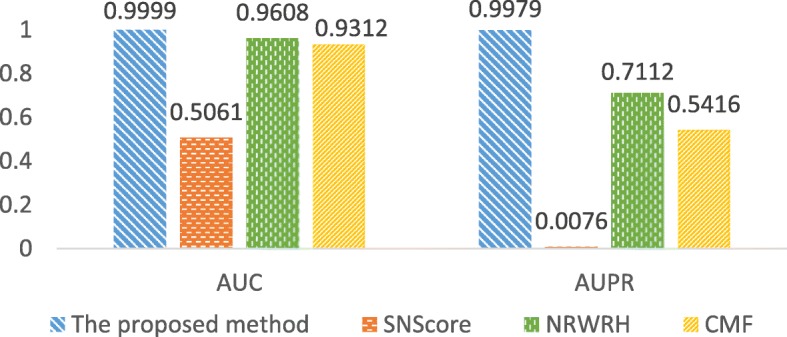
Comparison of the proposed method with other methods. NRWRH, Network-based Random Walk with Restart on Heterogeneous network. CMF, Collective Matrix Factorization

It is found that the proposed method outperforms the others significantly (Fig. 8). Although NRWRH and CMF achieve relatively high AUC, they perform much worse than the proposed method in terms of AUPR. In NRWRH, CMF and the proposed method, exactly the same data are used, including the pairwise associations among drugs, targets and diseases, as well as similarity matrices of drugs and targets. It is worth noting that CMF is very similar to the proposed method, but decomposes two-dimensional matrices of pairwise associations instead of three-dimensional tensors. The better performance of the proposed method indicates that the tensor structure might contribute to the prediction of triplet associations, especially in the comparison with CMF. One possible reason is that, the proposed method extracts the functional patterns from triplet associations, while the factor matrices extracted in CMF method only capture the pairwise functional patterns, and random walk on networks might loss some global topological information.

### Identification and validation of new predictions

By recovering missing values in association tensor *χ* using the derived factor matrices, i.e. *C*, *P* and *D*, we get a new association tensor $\widetilde {\chi }$ and the predicted score for each unobserved associations inside (Fig. 1).

**Fig. 9:**
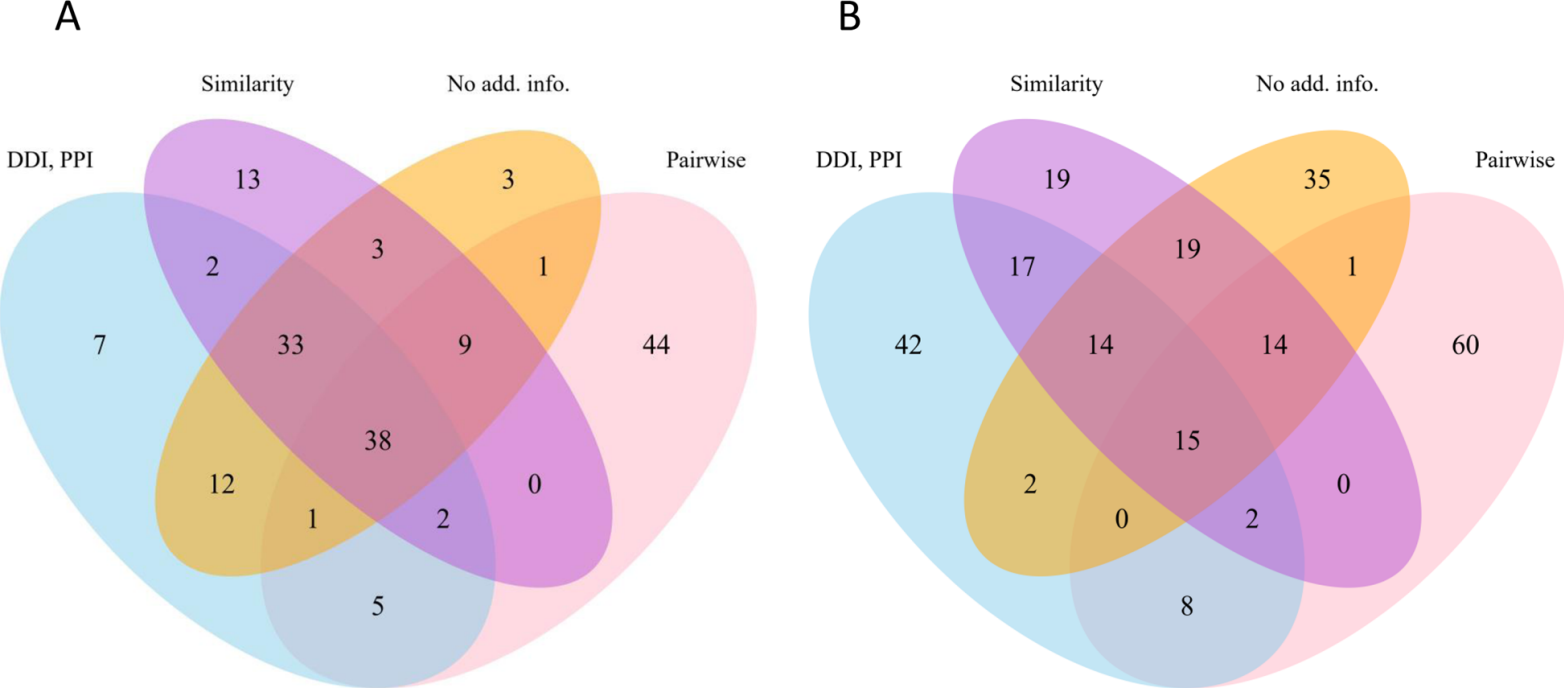
Illustration of prediction overlaps using different additional information. Overlaps in the top 100 predictions derived from *χ*^*tri*^ (**a**) and *χ*^*bi*^ (**b**) are included. No add. info., using no additional information. Pairwise, using pairwise associations as additional information

First, we compare the top predictions extracted from tensor decomposition with different additional information. Figure 9 shows the overlaps of predictions derived from *χ*^*tri*^ (a) and *χ*^*bi*^ (b). The decomposition of *χ*^*tri*^ has 38 predictions in common, while *χ*^*bi*^ has 15 in common using 4 different additional information settings. The top predictions of *χ*^*bi*^ diverse more since different additional information rate high scores for different triplets. It means that when there are more associations in a tensor, i.e. more information to infer from, more diverse associations might receive high prediction scores. However, we find that the overlap of predictions using similarity as additional information and using other additional information is consistently large in both *χ*^*tri*^ and *χ*^*bi*^, which means that similarity is more robust than the others. In addition, the predictions from decomposition using similarity and no additional information have the largest overlap, revealing that the result from these two settings are more similar, again supporting the fundamental assumption.

**Fig. 10:**
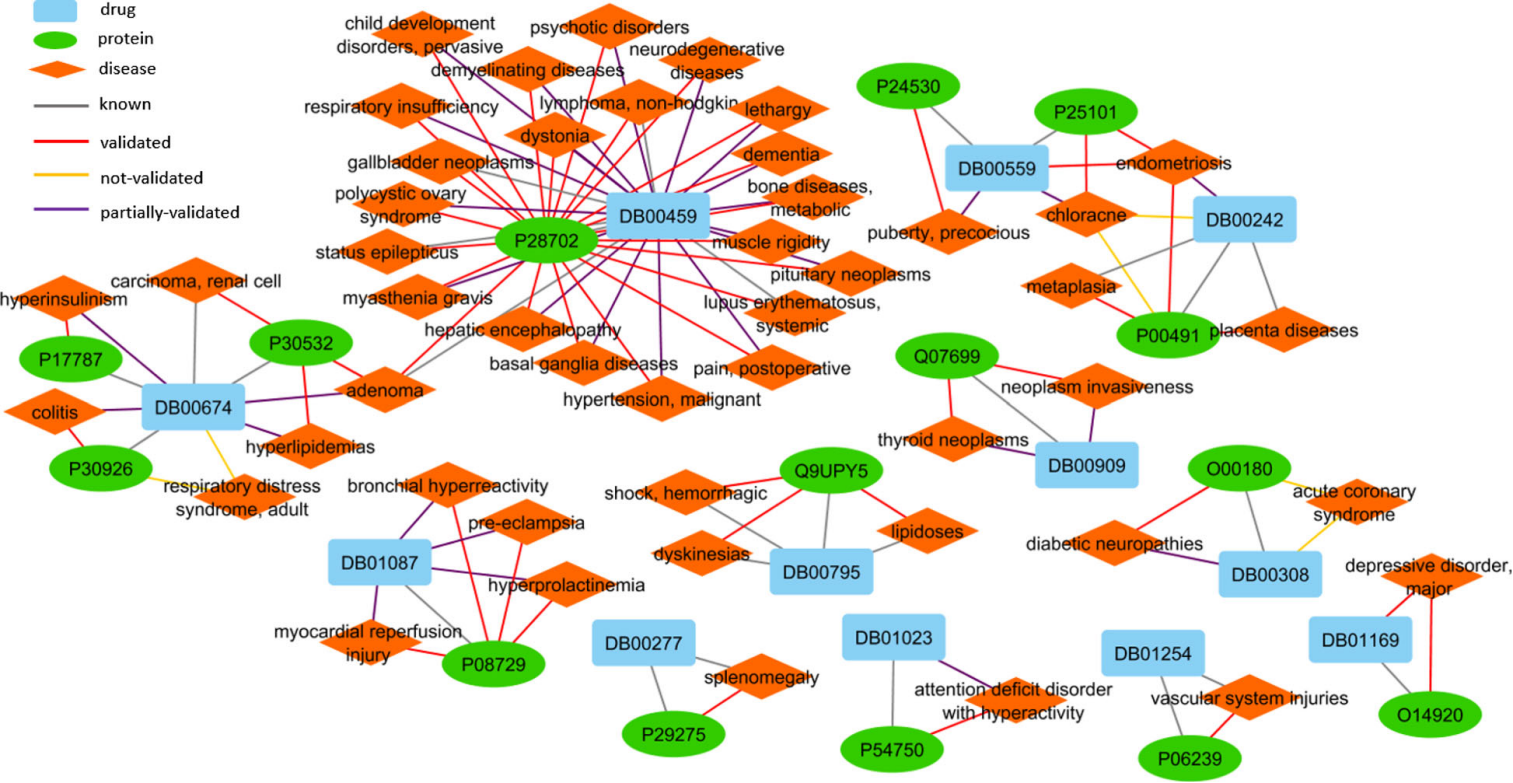
Network visualization of predicted and known pairwise associations in the top 50 triplet predictions. Blue rectangles, green ellipses and orange diamonds represent drugs, targets and diseases, respectively. Red lines are predictions validated by literature support. Purple lines are inferred by the corresponding drug-target associations and target-disease associations. Grey lines are known associations

Since the performance of decomposing *χ*^*bi*^ using similarity is the best, and the result is robust and very similar with using no additional information, we use the result of it for new prediction validation. The statistics of newly discovered pairwise associations in the top predictions is summarized in Table 2. We search for literature validation in both new version of Comparative Toxicogenomics Database (CTD 2017) and PubMed to validate the association predictions. We find support for most of the target-disease associations but only a few of drug-disease association predictions appeared in the top 50 predictions. The retrieved inference scores from CTD are summarized in (Additional file 10: Figure S10). The newly discovered and known associations in the top 50 predictions are visualized in Fig. 10. The grey lines and red lines represent known associations and new predictions, respectively. The purple lines are inferred with the assumption used in constructing *χ*^*bi*^. However, there are still three pairs of associations in yellow color that we find no support for, which deserves further study.

**Table 2:** Statistics of newly discovered pairwise associations in the top predictions

Associations	Top 50 predictions		Top 10,0000 predictions	
	Known	Unknown	Known	Unknown
Drug-target	50	0	8608	1392
Drug-disease	14	36	4130	5870
Target-disease	0	50	1392	8608

We find no new discovered drug-target interactions in the top 50 predictions. To investigate the ability of discovering new drug-target interactions of the proposed method, we validate the top 10 drug-target interaction predictions appeared in the top 10,000 triplet predictions (Table 2) by both literature search and computational docking. Five of them are supported by existing literature. There are two proteins without known 3D structure among the top 10 pairs, which cannot be used for computational docking. Since the predicted interaction of one of the two proteins has been validated by literature search, we take the 11th-ranked pair for validation in replacement of the pair without support. The support references and docking scores are summarized in Table 3. The docking pockets and poses of three pairs are illustrated in (Additional file 11: Figure S11). All of the above results indicate that the proposed method is able to discover new associations among drugs, targets and diseases based on their observed associations and similarity information.

**Table 3:** Supporting references and docking scores of the top-ranking drug-target predictions

Drug	Protein(Gene)	Reference/Docking score
Miglitol	SI	[45] / −6.4 kcal/mol
Tropicamide	CHRM5	CTD/ –
temsirolimus	FKBP1A	[46] / −10 kcal/mol
Tacrolimus	MTOR	[47] / −7.4 kcal/mol
Dopamine	ALDH2	CTD / −6.6 kcal/mol
Amifostine	NT5C2	−5.6 kcal/mol
Vigabatrin	GABBR2	−5.1 kcal/mol
Verapamil	KCNK1	−7.9 kcal/mol
Zonisamide	AQP1	−7.1 kcal/mol
Metyrapone	FDX1	−8.7 kcal/mol

### Analysis of latent factors

The latent factors derived from tensor decomposition characterize the associations and functional patterns of drugs, targets and diseases. Similar latent factors indicate similar behaviors. Thereby, it is interesting to study the latent factors to uncover the underlying behavioral characteristics.

#### Latent factors derived from different additional information

**Fig. 11:**
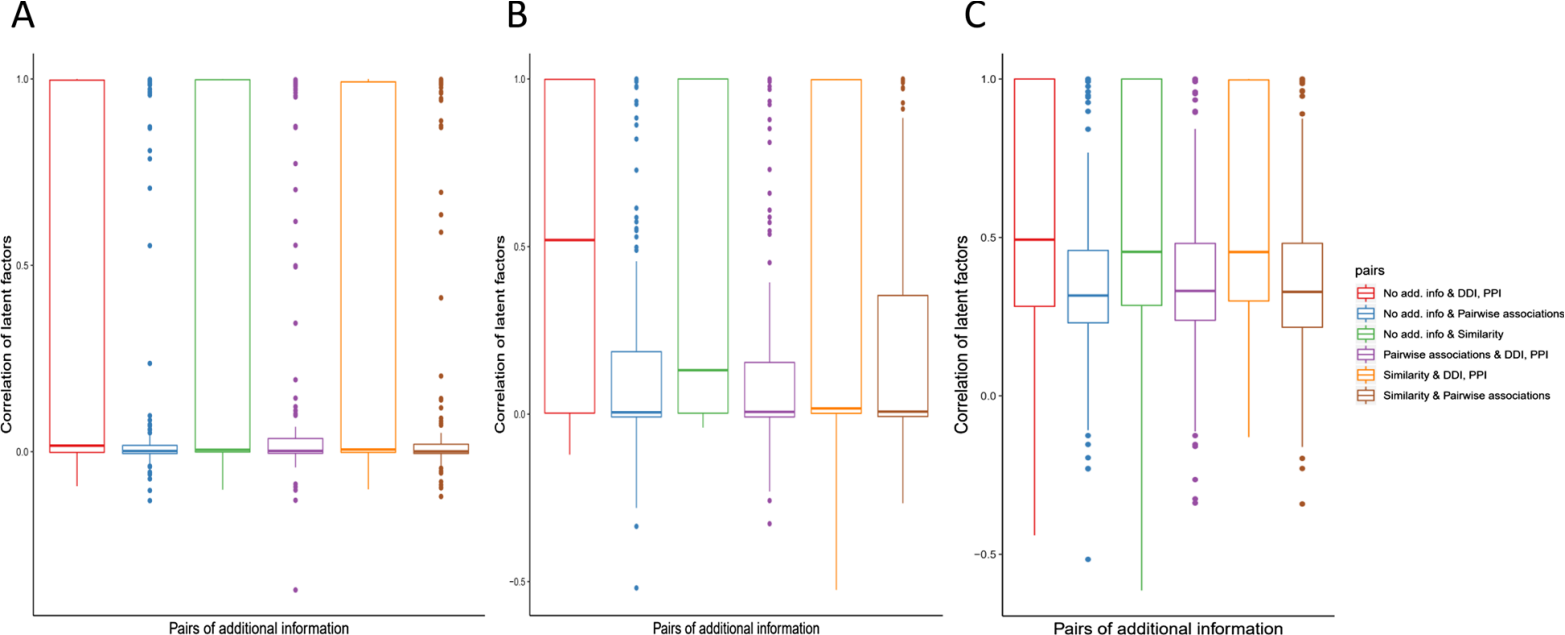
Correlation of latent factors derived from *χ*^*tri*^. Different kinds of additional information are used. **a** Latent factors of drugs. **b** Latent factors of targets. **c** Latent factors of diseases. No add. info, using no additional information

We compare the latent factors extracted from association tensor decomposition utilizing different additional information. Figure 11 and (Additional file12: Figure S12) illustrate the pairwise correlation of latent factors derived from different settings. The latent factors from similarity, as well as DDIs and PPIs show higher correlation with latent factors using no additional information. It is consistent with the previous finding that the predictions from using no additional information overlap the most with those extracted from similarity, DDIs and PPIs. One possible reason is that such additional information has strong relation with the functional behaviors of drugs, targets and diseases.

#### Clustering of drugs, targets and diseases using latent factors

We adopt Topological Data Analysis (TDA) to cluster the drugs, targets and diseases into functionally similar groups based on latent factors and investigate the properties of the groups [26] (Methods).

**Fig. 12:**
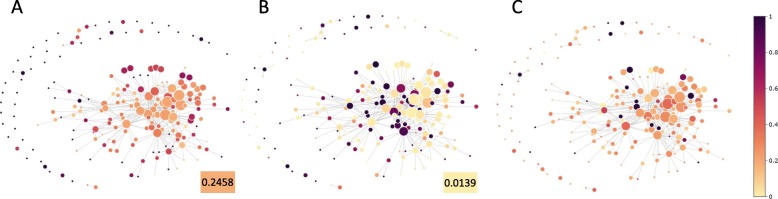
Topological data analysis of drugs. The nodes are clusters of drugs and the colors demonstrate the property of nodes. **a** Drug clustering vs. intra-cluster drug similarity. Darker color indicates higher similarity. **b** Drug clustering vs. Jaccard similarity of their interactions with targets. Darker color indicates higher Jaccard similarity. **c** Drug clustering vs. Jaccard similarity of their associations with diseases. Darker color indicates higher Jaccard similarity

For drugs, we compare their clusters with their structural similarity, similarity of their associations with targets and diseases (Methods), their super classes and their AHFS codes, respectively. Drug clusters are illustrated in Fig. 12, where nodes are drug clusters and colors indicate properties of the nodes. Compared to the average pairwise similarity of all drugs, which is 0.2458, drugs in a lot of clusters show higher intra-cluster chemical similarity (Fig. 12a). It means that many structurally similar drugs are clustered together due to their similar functions. On the contrary, there are some clusters with low intra-cluster chemical similarity, indicating that even though their chemical structures are very different, they have similar functional behaviors. For example, Amiloride and Diclofenac demonstrate very low similarity in terms of chemical structure. The efficacy of Amiloride includes diuretic and epithelial sodium channel blocker, while the efficacy of Diclofenac is analgesic, anti-inflammatory, antipyretic and COX inhibitor. These two drugs are clustered together by the latent factors, and we find that they share a common target Acid-sensing ion channel 1, reflecting their similar underlying functions.

The Jaccard similarity of drugs in terms of association with targets reflects their functional similarity with respect to drug-target interactions. The average target-association similarity of all drugs is 0.0139. Figure 12b demonstrates that some drugs with similar target associations are clustered together. Meanwhile, a lot of clusters show low similarity. The similarity distribution of drugs in terms of drug-disease associations is illustrated in Fig. 12c. Comparing Fig. 12b and 12c, it is found that, as a compensation, clusters of drugs with low intra-cluster similarity of target associations demonstrate relatively high similarity of intra-cluster disease associations.

In addition, we compare the clustering of drugs with their super classes and AHFS codes in DrugBank. A lot of drugs from the same super class or same AHFS class are clustered together in several nodes (Additional file 13: Figure S13). Classes with large number of drugs dominate more clusters in the network and occupy very high proportion in these clusters. In classes with a small number of drugs, most of the drugs are gathered in a few clusters. For example, there are 9 drugs from the class Nucleosides, nucleotides, and analogues in our dataset. According to (Additional file 13: Figure S13b), most of them are gathered in only two clusters.

**Fig. 13:**
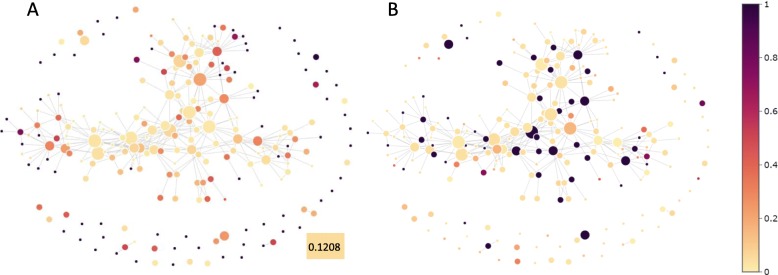
Topological data analysis of targets. The nodes are clusters of targets and the colors demonstrate the property of nodes. **a** Target clustering vs. intra-cluster target protein similarity. Darker color indicates higher similarity of target proteins. **b** Target clustering vs. Jaccard similarity of their associated GO terms. Darker color indicates higher Jaccard similarity

For targets, we compare the clustering result with their similarity of amino acid sequences and Gene Ontology (GO) annotations (Fig. 13). Similar to drugs, targets in the same cluster also show higher similarity compared to the average similarity, which is 0.1208. We collect GO annotations of the targets and calculate the Jaccard similarity of each pair of targets in terms of their associated GO terms (Methods). A lot of clusters receive very high similarity scores, indicating their similar functions, especially several clusters with more than 20 targets each. TDA of diseases demonstrates similar results with drugs, that diseases from the same class tend to appear in the same cluster, meaning that diseases with similar molecular mechanisms are clustered together (Additional file 14: Figure S14).

### Drug repositioning for cancer

To repurpose drugs for cancers, we apply the proposed method to the cancer subset, which is extracted from the same original dataset provided in [17] (Methods). We extracted 309 cancer subtypes in total, and keep all of the targets and diseases provided. Since some of the cancer subtypes are known to be targeted by their associated genes, we do not use any additional information, and construct the association tensor for cancers using the first strategy to avoid noise.

**Fig. 14:**
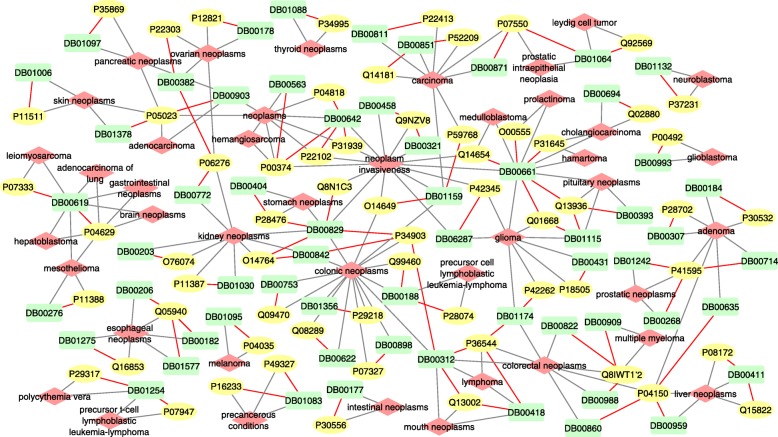
Visualization of top 100 predictions in cancer drug repositioning. Green rectangles are drugs. Yellow ellipse are targets. Red diamonds are cancers. Red lines are new predictions. Grey lines are known associations

All of the predicted drug-cancer pairs ranking in the top 100 are involved in validation. We visualize the network of the top predictions in Fig. 14. After searching for the drug-cancer association predictions in PubMed, we find literature support for 23 out of 25 pairs. For example, Prednisolone is known with its efficacy of anti-inflammatory, congenital adrenal hyperplasia, psoriatic arthritis, pemphigus, etc, and shows no anti-tumor effect in KEGG DRUG. It ranks at top of our predictions by its association with colorectal neoplasms. We find several studies in recent two years reporting the use of Prednisolone in colon carcinoma treatment [27, 28]. We also discover associations between Etacrynic acid and adenocarcinoma [29], Etacrynic acid and pancreatic neoplasms [29], Temsirolimus and neoplasm invasiveness [30], etc. All of the drug-cancer associations with high prediction scores as well as their supporting references are listed in the (Additional file 15: Table S1).

Furthermore, we also find associations between drugs and cancer subtypes not indicating new therapeutic use, but showing adverse effects. For example, in our prediction, Leflunomide (LEF) is associated with pancreatic neoplasms, and we find that LEF is reported to increase the risk for pancreatic neoplasms in several studies [31]. We also validate several adverse effect predictions by literature search, including Malathion and kidney neoplasms [32], Magnesium cation and skin neoplasms [33], as well as Valsartan and intestinal neoplasms [34].

**Fig. 15:**
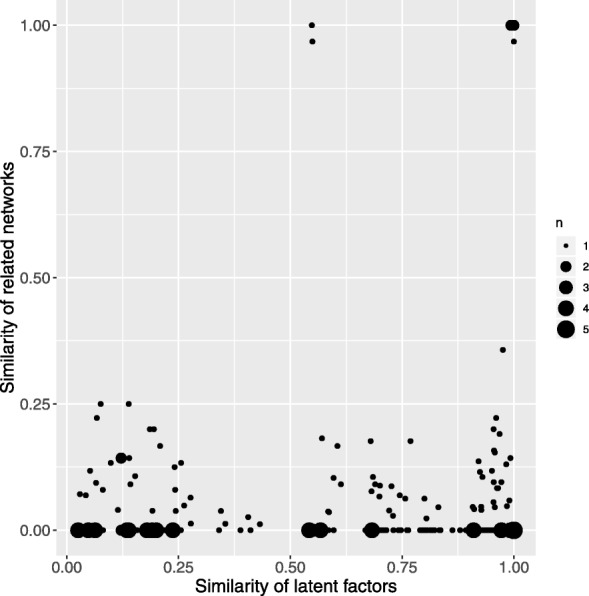
Comparison of the similarity of cancers in terms of related perturbed molecular networks (y-axis) and latent factors (x-axis). The size of nodes indicates the number of cancers with approximate similarity, shown as the legend on the right

In addition, we investigate the latent factors of drugs, targets and cancer subtypes. KEGG NETWORK is a database capturing knowledge on diseases and drugs in terms of perturbed molecular networks [35]. So far, it only provides networks about cancers. The perturbed molecular networks of cancers reflect their mechanisms from genome variation aspect. We retrieve the associated networks of cancer subtypes involved in our dataset from KEGG NETWORK and get network information for 28 of them. Then we compare similarity of cancer subtypes in terms of their latent factors and their associated perturbed molecular networks. Cosine similarity of the latent factors and Jaccard similarity of associated networks are calculated for each pair of cancer subtypes. Among 28 cancer subtypes, only 9 pairs receive high similarity scores of associated networks, while most of them get very low similarity below 0.25. For the 9 pairs with high network similarity, the latent factors of 7 pairs also demonstrate very high similarity, while the other 2 have medium similarity (Fig. 15). For the other pairs with very low similarity of perturbed molecular network, the latent factors show very diverse similarity. The analysis supports that the latent factors derived from association tensor decomposition is able to capture the underlying mechanisms of cancers from their associations with drugs and targets.

**Fig. 16:**
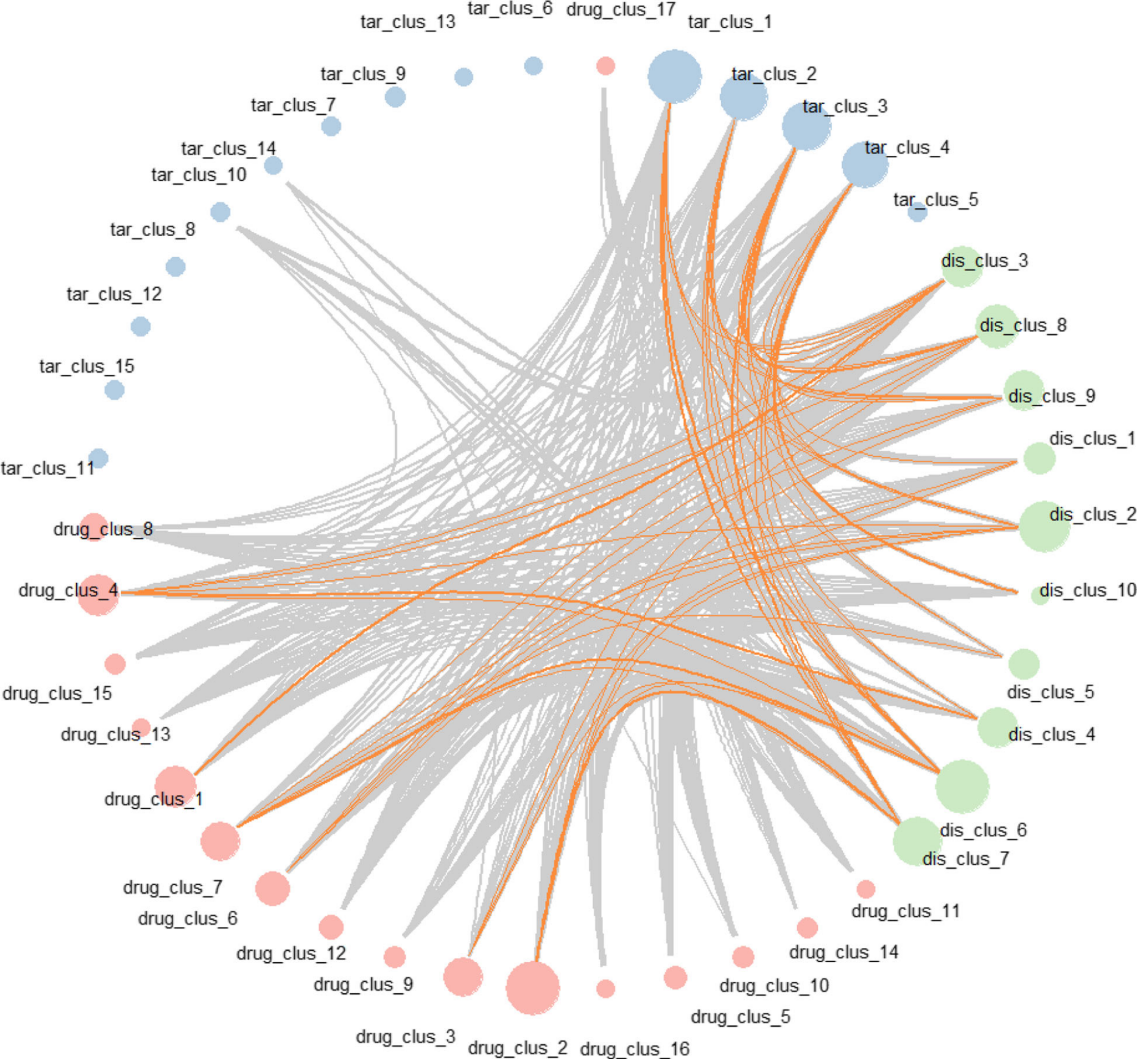
Associations between clusters of drug, targets and cancers. Red circles, blue circles and green circles represent drug clusters, target clusters and cancer clusters, respectively. The Grey lines and orange lines indicate the known and new discovered associations among clusters, respectively. The width of the lines indicates the number of associations between the clusters. The size of the nodes indicates the number of drugs/targets/cancers in the clusters

We cluster the drugs, targets and cancer subtypes according to their latent factors (Fig. 16). A lot of small clusters of targets, which are clustered far away from the others, have no association with the drugs and cancer subtypes, such as SSTR1, PCCA, MDH1, etc. All of the clusters of drugs and cancer subtypes have associations with other clusters, even if the cluster is very small. Some clear patterns of associations between clusters are found. For example, for known associations (grey lines), targets in cluster 10 have stronger associations with cancer subtypes in cluster 3, while drugs in cluster 6 have more associations with cancer subtypes in cluster 2. For new predictions (orange lines), strong associations between particular clusters are also obvious, such as drug cluster 1 with cancer cluster 3, drug cluster 7 with cancer cluster 6, drug cluster 2 with cancer cluster 7, and target cluster 1 with cancer cluster 6. The results indicate that there exist specific association patterns among the clusters. For example, a kind of drugs tends to be associated with particular kind of targets and cancer subtypes. This finding suggests that the successful rate for drug repositioning might be improved if we repurpose drugs to cancer subtypes which are selected from two clusters with very strong associations.

## Discussion

Drug discovery is the process to discover or design new drugs for some purposes. The conventional method of drug discovery is time-consuming and costly, which usually takes 13-15 years and costs 2-3 billions to develop a new drug. Drug repositioning aims to discover new therapeutic uses for existing drugs, which takes advantage of drugs that have passed a significant number of toxicity and other tests. A variety of computational methods have been proposed for drug repositioning, which is much more efficient and effective, including machine-learning-based methods [6, 9], matrix decomposition-based methods [10, 13] and network-based methods [15, 16]. Most of the studies investigate pairwise associations among drugs, targets and diseases. However, pairwise associations cannot uncover the whole picture of underlying mechanisms. For examples, some indications of drugs have been discovered without knowing the corresponding targets [14]. In this paper, we have studied the triplet associations among drugs, targets and diseases to uncover the triangular relationships among them.

The triplet associations are modelled as a tensor and new associations are discovered by tensor decomposition. Different strategies are used to construct the association tensor and we find that the strategy involving inferred associations achieves better performance. One possible reason is that the tensor constructed by the this strategy contains more information of the drugs, targets and diseases. In addition, different kinds of additional information are used, including similarity of drugs and targets, DDIs and PPIs, as well as pairwise associations. As expected, using similarity as additional information improves the performance the most when the number of latent factors is small, demonstrating the effectiveness of similarity. Since the similarity information of drugs and targets, as well as DDIs and PPIs are widely used in this field, we investigate whether they could also contribute to the triplet association study in this paper. However, there are some other kinds of additional information to be further studied which might perform better, e.g. network topological information. Similarity of diseases could also be involved to provide more additional information. Furthermore, since sparsity is crucial in recommendation systems which are analogous to our problem, we investigate the effects of sparseness, association enrichment and similarity of association patterns of the tensor on the performance. It is found that lower association enrichment and fewer similar drugs, targets or diseases in terms of associations patterns, instead of higher sparseness of the tensor, hurt the performance a lot, indicating that in this problem, the quality of data plays a more important role than the amount of information involved. It is worth to identify other factors related to the performance.

The proposed method is compared to three baseline methods which investigate pairwise associations among drugs, targets and diseases. The proposed method outperforms the others in both AUC and AUPR, which indicates the advantage of the proposed method in predicting triplet associations compared to the pairwise-focusing methods. It is found that the predictions derived using similarity as additional information overlap the most with those derived from the single association tensor, which supports the widely accepted assumption in the community that similar drugs bind to similar targets. Most of the predictions ranking in the top 50 have been validated by literature search. For example, one of the top-ranking triplet prediction is the association among Galantamine, neuronal acetylcholine receptor subunit beta-4 (CHRNB4) and colitis, where the interaction between Galantamine and CHRNB4 is known before the prediction. Evidence for the anti-colitic effects of Galantamine is reported in a recent study [36]. Adverse effect associations between drugs and diseases are also found in the top-ranking predictions, such as the triplet association among Arsenic trioxide, inhibitor of nuclear factor kappa-B kinase subunit beta (IKBKB) and major depressive disorder. The association information between Arsenic trioxide and IKBKB is included as known association in DTD subset. It is reported that Arsenic down-regulates expression of genes responsible for long-term potentiation and depression [37]. In addition, some top-ranking drug-target pairs have been validated by literature search or computational docking, such as Zonisamide vs. AQP1, Verapamil vs. KCNK1, as well as Metyrapone vs. FDX1.

The latent factors extracted from association tensors capture the behavioral patterns and underlying molecular mechanisms of drugs, targets and diseases. The latent factors derived from similarity, DDIs and PPIs have higher correlation with that of using no additional information, indicating that the underlying patterns reflected in similarity of drugs and targets, as well as DDIs and PPIs, are more similar to their association patterns. The drugs, targets and diseases are clustered into functional groups based on the latent factors and some intra-cluster properties are found, for example, drugs and targets clustered together show higher structural and functional similarity, and diseases from the same class tend to appear in the same cluster. However, the number of drugs, targets and diseases in DTD subset is limited to 549, 424 and 340, respectively. Large-scale analysis is more interesting and is worth of study in the future, which might discover more triplet associations.

After the investigation of a variety of diseases, we focus on cancers and apply the proposed method to a cancer subset. New associations between drugs and cancer subtypes are identified. It is found that some of these new discovered associations indicate new therapeutic uses of drugs, including the anti-tumor effect of Prednisolone, while others demonstrate adverse effects, for example, LEF might increase the risk for pancreatic neoplasms. When investigating the latent factors of cancer subtypes, we find that most of the pairs of cancer subtypes with high similarity of KEGG NETWORK associations receive similar latent factors. It supports that the proposed method is able to capture the characteristics of cancer mechanisms. In the in-depth study of the clusters of drugs, targets and cancer subtypes, we find clear behavioral patterns between particular clusters. It provides a new guiding framework for drug repositioning: We can repurpose drugs to diseases that come from two clusters with strong associations, instead of inferring new drug-disease associations only by finding drugs with similar chemical structure to the disease-associated drugs, since in many cases, functional similar drugs have very different chemical structures.

## Conclusion

In this paper, we have proposed a novel framework for drug repositioning based on decomposing the triplet association tensors of drugs, targets and diseases. The proposed method is able to recover missing associations and predict unobserved triplet associations. Most of the top ranked predictions have been validated by literature search and computational docking. The proposed method have outperformed some baseline methods in both AUC and AUPR. The drugs, targets and diseases have been clustered into functional groups based on the extracted factor matrices. We have also applied the proposed method to a cancer dataset and discovered strong associations between particular clusters, which provides a new guiding framework for drug repositioning.

## Methods

### Data collection

We use the dataset in a recently published paper by Luo et al. [17]. They have collected 708 drugs from DrugBank [38], 1,512 targets from Human Protein Reference Database (HPRD) [39] and 5,603 diseases from Comparative Toxicogenomics Database (CTD) (2013) [40]. The corresponding drug-target associations and DDIs are collected from DrugBank. The corresponding PPIs are collected from HPRD. The drug-disease associations and target-disease associations are collected from CTD.

We construct two sub-datasets from the original dataset, which are the DTD subset and the cancer subset. For the DTD subset, in order to ensure the data quality and to reduce sparseness, we collect the most informative drugs, targets and diseases with relatively more associations. We remove the drugs and targets with no association, as well as diseases with less than 300 target-disease associations or 100 drug-disease associations, since the average number of associated targets of a disease is more than the average number of associated drugs of a disease, resulting in 549 drugs, 424 targets and 340 diseases remained. We extract the corresponding associations, DDIs and PPIs of the remaining drugs, targets and diseases. Details can be found in our previous paper [41]. For the cancer subset, we select all cancer subtypes from the original dataset referring to the disease classification in CTD, and keep all of the drugs and targets. The corresponding associations, DDIs and PPIs are collected. As a result, there are 309 cancer subtypes, 708 drugs and 1512 targets in the cancer subset. The similarity between drugs is calculated by the Tanimoto coefficient of the product-graphs of the chemical structures [17]. The similarity between proteins is the Smith-Waterman score based on their amino acid sequences.

### Construction of the drug-target-disease association tensors

Since it is hard to collect triplet associations directly, the association tensors are constructed from the pairwise associations. We develop two strategies with different levels of tolerance in tensor construction. In the first strategy, if drug A interacts with target B, target B is associated with disease C, meanwhile, drug A is associated with disease C, then we believe that the triplet association among drug A, target B and disease C exists, represented by one at the corresponding entry in the tensor. Otherwise, the triplet association is regarded as unknown and represented by zero in the corresponding entry. The second strategy is based on weaker conditions under an assumption that, if drug A is associated with target B, and target B is associated with disease C, then it can be inferred that drug A is associated with disease C [42]. Thereby, in the second strategy, we regard the triplet association as an observation if both of the corresponding drug-target interaction and target-disease association have been discovered. By applying the two strategies on the DTD subset, we construct two triplet association tensors with different sparseness, which are *χ*^*tri*^ with ∼0.33*%* observations (constructed by the first strategy) and *χ*^*bi*^ with ∼0.76*%* observations (constructed by the second strategy), respectively. For the cancer subset, we only adopt the first strategy to avoid noise and construct a tensor *χ*^*ca*^ with ∼(1.37*E*−2)*%* observations.

### Predicting triplet associations among drugs, targets and diseases based on tensor decomposition

In this paper, the problem of triplet association prediction is modeled as tensor completion (Fig. 1). A three-dimensional association tensor *χ* is factorized into three matrices, called factor matrices, which capture the functional patterns of drugs, targets and diseases, respectively. By multiplying the factor matrices, another tensor $\tilde {\chi }$ is generalized, which contains the approximation of the observations in *χ* and new predictions recovered from the functional patterns. However, due to our limited knowledge, the association tensor *χ* constructed from known associations is very sparse, which means that the information of the drugs, targets and diseases that can be inferred from is deficient, making it hard to extract the truly underlying patterns. To integrate more useful information, different kinds of additional information, e.g. similarity information, DDIs and PPIs, are involved. The model of the proposed method is as follows:

1 $$\begin{array}{@{}rcl@{}} \min_{C,T,D,d_{i}} & & \omega_{main}\|\chi-\llbracket {C,T,D}\rrbracket \|_{F}^{2} \\ &+& \Sigma_{i=1}^{M} \omega_{i}\|S_{i}-A_{i}d_{i}B_{i}^{T}\|_{F}^{2} \\ &+& \omega_{reg}(\|C\|_{F}^{2} + \|T\|_{F}^{2} + \|D\|_{F}^{2})  \\ &+& \omega_{reg}\Sigma_{i=1}^{M}\|d_{i}\|_{F}^{2} \end{array} $$

2 $$\begin{array}{@{}rcl@{}} \min_{C,T,D,d_{ccs},d_{pps}} & &\omega_{main}\|\chi\,-\,\llbracket {C,T,D}\rrbracket \|_{F}^{2}\\ &+& \omega_{1}\|S_{ccs}\-Cd_{ccs}C^{T}\|_{F}^{2} + \omega_{2}\|S_{pps}\,-\,Td_{pps}T^{T}\|_{F}^{2} \\ &+& \omega_{reg}(\|C\|_{F}^{2} + \|T\|_{F}^{2} + \|D\|_{F}^{2}\\ &+& \|d_{ccs}\|_{F}^{2} + \|d_{pps}\|_{F}^{2}) \end{array} $$

3 $$\begin{array}{@{}rcl@{}} \min_{C,T,D,d_{ddi},d_{ppi}} & &\omega_{main}\|\chi\,-\,\llbracket {C,T,D}\rrbracket \|_{F}^{2} + \omega_{1}\|S_{ddi}\\&-&Cd_{ddi}C^{T}\|_{F}^{2} \,+\, \omega_{2}\|S_{ppi}\,-\,Td_{ppi}T^{T}\|_{F}^{2} \\ &+&\omega_{reg}(\|C\|_{F}^{2} + \|T\|_{F}^{2} + \|D\|_{F}^{2}\\ &+& \|d_{ddi}\|_{F}^{2} + \|d_{ppi}\|_{F}^{2}) \end{array} $$

4 $$\begin{array}{@{}rcl@{}} \min_{C,T,D,d_{ct},d_{cd},d_{td}} & & \omega_{main}\|\chi-\llbracket {C,T,D}\rrbracket \|_{F}^{2} + \omega_{1}\|S_{ct}\\&-&Cd_{ct}T^{T}\|_{F}^{2} + \omega_{2}\|S_{cd}-Cd_{cd}D^{T}\|_{F}^{2} \\&+& \omega_{3}\|S_{td}-Td_{td}D^{T}\|_{F}^{2} \\ &+& \omega_{reg}(\|C\|_{F}^{2} + \|T\|_{F}^{2} + \|D\|_{F}^{2}\\ &+& \|d_{ct}\|_{F}^{2} + \|d_{cd}\|_{F}^{2}+ \|d_{td}\|_{F}^{2}) \end{array} $$

where ∥.∥ is Frobenius norm. *χ* is an association tensor. *C*, *T* and *D* are the factor matrices of drugs, targets and diseases, respectively. *S*_*i*_ is a matrix for particular additional information, while *A*_*i*_ and *B*_*i*_ are the factor matrices of *S*_*i*_. The number of additional information matrices used in the model is represented by *M*. In order to integrate information from different sources, common factor matrices are shared between the association tensor and additional information, which means that both *A*_*i*_ and *B*_*i*_ are one of *C*, *T* and *D* in our cases. Equations of models using particular additional information are discussed below. Since there might be different scales in different data sources, we use *d*_*i*_ to model the scaling difference. In general, the first line of the equation minimizes the difference between *χ* and $\tilde {\chi } (\tilde {\chi } = \llbracket {C,T,D}\rrbracket)$, which is recovered by the factor matrices. The second part minimizes the error of decomposing the additional information matrices. The third part aims to minimize the norm of the factor matrices in order to avoid over-fitting. *ω*_*main*_,*ω*_*i*_ and *ω*_*reg*_ are weights controlling the importance of each part.

In this paper, we use three kinds of additional information, including (1) the similarity between the drugs and targets (Eq. 2), due to the widely accepted assumption that similar drugs might interact with similar targets, (2) DDIs and PPIs (Eq. 3), which reflect the functional patterns of drugs and targets, and (3) pairwise associations among drugs, targets and diseases (Eq. 4), since some pairwise associations are lost in the tensor construction stage. The equations of decomposition using different additional information differ slightly (Eqs. 2–4), where *S*_*ccs*_ and *S*_*pps*_ are similarity matrices of drugs and targets, respectively. *S*_*ddi*_ and *S*_*ppi*_ are DDI and PPI matrices, respectively. *S*_*ct*_,*S*_*cd*_ and *S*_*td*_ are matrices of drug-target interactions, drug-disease associations and target-disease associations, respectively. *d*_*ccs*_ is the scaling difference between triplet associations and drug similarity matrix, while *d*_*pps*_ is the scaling difference between triplet associations and target similarity matrix. *d*_*ddi*_ and *d*_*ppi*_ are the scaling differences between triplet associations with DDI and PPI matrices, respectively. *d*_*ct*_,*d*_*cd*_ and *d*_*td*_ model the scaling difference between the pairwise associations and triplet associations. It is worth noting that the decomposition of similarity matrices, as well as DDI and PPI matrices, can be symmetric non-negative matrix factorization (NMF). In addition, when using pairwise associations as additional information, for each triplet association in the test set, all of the three related pairwise associations are removed by setting the corresponding values to zero in *S*_*ct*_,*S*_*cd*_ and *S*_*td*_. For example, if *χ*_*ijk*_ is in the test set, then $S_{ct_{ij}}, S_{cd_{ik}}$ and $S_{td_{jk}}$ are set to zero in the pairwise association matrices.

### Generation of random tensors for sparsity study

Since the triplet association tensors are very sparse, it is of interest to investigate whether the sparseness affects the performance of tensor decomposition. To construct random tensors with same size with *χ*^*tri*^ and *χ*^*bi*^, we first randomly select the same number of drugs, targets and diseases, which are 549, 424 and 340, respectively, from the original dataset [17]. Then random association tensors are constructed using the two strategies, respectively. By repeating this process five times, 5 pairs of random association tensors with different groups of drugs, targets and diseases, as well as different sparseness, are constructed. The proportion of observations in the random tensors are summarized in Table 1.

### Enrichment and similarity of triplet associations

In an association tensor *χ*, we define the association enrichment of drug *i*, represented by $E_{c_{i}}$, as the number of all corresponding observed triplet associations of the drug in *χ*, which is $E_{c_{i}}=\Sigma _{j=1}^{J}\Sigma _{k=1}^{K}\chi _{ijk}$, where *J* and *K* are the total number of targets and diseases in *χ*, respectively. Similarly, the association enrichment of target *j* and disease *k* is $E_{t_{j}}=\Sigma _{i=1}^{I}\Sigma _{k=1}^{K}\chi _{ijk}$ and $E_{d_{k}}=\Sigma _{i=1}^{I}\Sigma _{j=1}^{J}\chi _{ijk}$, respectively, where *I* is the total number of drugs in *χ*. The association enrichment of drugs, targets and diseases reflect the amount of useful interactive information of them, which might has an impact on the veracity of the extracted functional patterns, and further affects the accuracy of the predictions.

In order to calculate the similarity between drugs in terms of their triplet associations, we first flatten the tensor into a matrix by the first dimension, where each row is a vector representation indicating all triplet associations of a drug. Similarly, the vector representation of all triplet associations of each target and each disease is derived by flattening the tensor by its second and third dimension, respectively. Then Jaccard similarity is calculated between drugs, targets and diseases based on their association vectors by equation 5.

5 $$\begin{array}{@{}rcl@{}} S_{ij} =1-\frac{|(v_{ik}\neq v_{jk})\cap ((v_{ik}\neq 0)\cup (v_{jk}\neq 0))|}{|(v_{ik}\neq 0)\cup (v_{jk}\neq 0)|}  \end{array} $$

where *S*_*ij*_ is the similarity of association patterns between drug/target/disease *i* and *j*, *v*_*i*_ and *v*_*j*_ are the corresponding vector representation of triplet associations.

### The baseline methods

In this paper, we compare the proposed method to three methods, which are SNScore, NRWRH and CMF. SNScore adopts the number of in-between nodes of two nodes to calculate the probability of their connection. NRWRH is proposed to infer drug-target interactions using random walks on a heterogeneous network. In order to involve diseases into the method, it is generalized to networks with three different kinds of nodes instead of the original two. A heterogeneous network including associations among drugs, targets and diseases, as well as similarity between drugs and targets is constructed (Additional file 9: Figure S9a) using the DTD subset. CMF is the two-dimensional version of the proposed method, which investigates pairwise associations modeled by 2D matrix factorization. In CMF, three pairwise association matrices together with the similarity additional information from DTD subset are factorized collectively (Additional file 9: Figure S9b). Thus, NRWRH, CMF and the proposed method are performed on exactly the same dataset.

### Evaluation of the proposed method and baselines

*The proposed method* The proposed method is evaluated by AUC and AUPR under 10-fold cross-validation. In the experiments investigating the performance of the proposed method (Results), we find that the performance of decomposing *χ*^*bi*^ is better than that of decomposing *χ*^*tri*^ (Fig. 3). In addition, using similarity as additional information for *χ*^*bi*^ achieves the highest AUC and AUPR (Fig. 4), especially when setting the number of latent factors to 250 (Fig. 2). Therefore, the above setting, where *χ*^*bi*^ is decomposed together with the similarity matrices into 250 latent factors, is used in the comparison.

*SNScore, NRWRH and CMF* The baseline methods are proposed to infer pairwise associations instead of triplet associations. The ability of predicting triplet associations of the baseline methods are evaluated by the following steps: (1) Same with the proposed method, a subset (10%) of triplet associations are randomly selected from the association tensor (*χ*^*bi*^) as test samples, which is represented by *T*_*test*_. (2) The triplet associations in *T*_*test*_ are projected to corresponding pairwise associations. For example, a test sample $\chi _{ijk}^{bi} \in T_{test}$ is projected to three pairwise associations, including the interaction between drug *i* and target *j*$(S_{ct_{ij}})$, the association between target *j* and disease *k*$(S_{td_{jk}})$, as well as the association between drug *i* and disease *k*$(S_{cd_{ik}})$. (3) One of the three projected pairwise associations of each test sample is randomly masked by setting the corresponding value to zero in the pairwise association matrices in CMF or removing the corresponding edge from the heterogeneous network in NRWRH. (4) Running the baseline methods to infer the probability of the masked pairwise associations. (5) The inferred score $(P_{drug\_tar\_dis})$ of each test sample is calculated by the following equation:

6 $$\begin{array}{@{}rcl@{}} P_{drug\_tar\_dis} = P_{drug\_tar} \times P_{tar\_dis}  \end{array} $$

where $P_{drug\_tar}$ is the inferred probability or known binary association between the corresponding drug and target. $P_{tar\_dis}$ is the inferred probability or known binary association between the corresponding target and disease. (6) The AUC and AUPR are calculate based on the test samples and their inferred scores. (7) Steps (1)-(6) are repeated 10 times, and the average AUC and AUPR are calculated and used in comparison.

For SNScore, since the source code is not available, we only search for the pairwise associations to be inferred in the above steps on SNScore platform to calculate the inferred score for test samples. For NRWRH, we use grid search to optimize the parameters, including the value of maximum iteration, restart probability and transition probability, which are finally set to 400, 0.5 and 0.2, respectively. For CMF, we check the performance with different number of latent factors and find that the performance does not vary much when the number of latent factors is in range of 150 to 250 (Additional file 16: Figure S15). We finally set the number of latent factors in CMF method to 160 in the comparison study.

### Topological Data Analysis

TDA is proposed to extract information from high-dimensional data, which is often incomplete and noise, and hard to visualize. It performs dimensionality reduction and provides a way to study the shape of data and manifolds. Mapper [26], a TDA method, constructs a graph from data which is able to reflect important connectivity features and is easy to visualize. In the graph constructed by Mapper, each node is a cluster of items and each edge between two nodes means that the two clusters share at least a common item. We adopt Mapper to cluster the drugs, targets and diseases separately, and investigate the properties of the clusters. Since the latent factors reflect the association patterns of the three kinds of entities, the clusters indicate their functional groups. We use two-dimensional scaling projection to project the latent factors and use k-means for clustering. For drugs and targets, to determine the settings of the major parameters in Mapper, including the number of clusters, the resolution and the overlap of clusters, we use the assumption that structurally similar drugs/targets have similar functions as a measurement. Experiments show that the more clusters, the higher intra-cluster similarity. However, it is meaningless to study clusters with only a little drugs/targets each. Thus, we balance the intra-cluster similarity with the three parameters to derive less clusters with relatively high intra-cluster similarity. The number of clusters of drugs and targets are 9 and 8, respectively. The resolution of drugs and targets are 8 and 11, respectively. The overlap is set to 0.4. For diseases, without any assumption and measurement, we use the default setting of resolution, i.e. 10, and keep the selected setting of overlap for drugs and targets, i.e. 0.4. Since the number of diseases is less than that of drugs and targets, we set the number of clusters in local clustering for diseases to 5.

In the result graphs, each node represents a cluster of drugs/targets/diseases, and each edge indicates that the two nodes share at least a common drug/target/disease. The color of nodes demonstrates different properties of clusters. For drugs, we use color distribution to illustrate the intra-cluster structural similarity, Jaccard similarity of their associations, as well as class distributions on the graph. The intra-cluster structural similarity is calculated by averaging the chemical similarity of each drug pairs in the cluster. The target/disease associations of each drugs is represented by a one-hot vector. The Jaccard similarity of target/disease associations of a pair of drugs is calculated by equation 5. The super classes and AHFS codes of drugs are collected from DrugBank. For targets, The sequence similarity of target proteins and Jaccard similarity of GO annotations are displayed on the target clustering graph by colors. The GO annotations are collected from Gene Ontology [43, 44]. The Go annotations of each target is represented by a one-hot vector. The Jaccard similarity of GO terms of a pair of targets is calculated by equation 5. For diseases, the distribution of disease classes over the clusters are illustrated. The classes of diseases are collected from CTD.

### Clustering of drugs, targets and cancer subtypes in cancer subset and associations between clusters

We cluster the drugs, targets and cancer subtypes using their latent factors by hierarchical clustering. We set the number of clusters of drugs, targets and cancer subtypes to be 17, 15 and 10, respectively. Then the hierarchical clustering is cut by the determined number of clusters. We construct a graph (Fig. 16) illustrating the associations between the clusters. The nodes represent different clusters and the lines represent the associations between the clusters. If a drug in one cluster is associated with a disease in another cluster, then these two clusters are connected by a line. The width of a line indicates the number of associations between the corresponding clusters. Thus, we can find not only the existence of associations, but also the strength of them.

## Supplementary information

**Additional file 1** Figure S1. Performance of decomposing *χ*^*tri*^ with different additional information.

**Additional file 2** Figure S2. Performance of decomposing the five random tensors constructed by the second strategy.

**Additional file 3** Figure S3. Boxplot of association enrichment in random tensors constructed by the second strategy.

**Additional file 4** Figure S4. Similarity of triplet associaiton patterns of drug pairs (a), target pairs (b) and disease pairs (c) in the five random tensors constructed by the second strategy.

**Additional file 5** Figure S5. Boxplot of similarity of triplet associaiton patterns of drug pairs (a), target pairs (b) and disease pairs (c) in the five random tensors constructed by the first strategy.

**Additional file 6** Figure S6. Boxplot of similarity of triplet associaiton patterns of drug pairs (a), target pairs (b) and disease pairs (c) in the five random tensors constructed by the second strategy.

**Additional file 7** Figure S7. Boxplot of association enrichment in random tensors constructed by the second strategy, compared to that of *χ*^*bi*^.

**Additional file 8** Figure S8. Boxplot of association enrichment in random tensors constructed by the first strategy, compared to that of *χ*^*tri*^.

**Additional file 9** Figure S9. Illustration of the baseline methods.

**Additional file 10** Figure S10. Distribution of retrieved inference scores in CTD.

**Additional file 11** Figure S11. Docking poses for three top-ranking drug-target predictions.

**Additional file 12** Figure S12. Correlation of latent factors derived from *χ*^*bi*^.

**Additional file 13** Figure S13. Topological data analysis of drugs in terms of drug classes.

**Additional file 14** Figure S14. Topological data analysis of diseases.

**Additional file 15** Table S1. Top-ranking predictions of drug indications for cancers and corresponding literature support.

**Additional file 16** Figure S15. Performance of CMF with different number of latent factors.

## Data Availability

Data used in this paper come from the study [17], which can be downloaded from https://github.com/luoyunan/DTINet. All other data that support the results of this study are available from the corresponding author upon request. Tensorlab is used in this study for tensor decomposition (https://www.tensorlab.net/).

## Abbreviations

AUC: Area under the receiver operating characteristic curve
AUPR: Area under precision-recall curve
CMF: Collective matrix factorization
CS: Core signature
CTD: Comparative toxicogenomics database
DDI: Drug-drug interaction
DO: Disease ontology
DTD: Drug-target-disease
FDA: Food and drug administration
GO: Gene ontology
HPRD: Human protein reference database
LEF: Leflunomide
NMF: Non-negative matrix factorization
NRWRH: Network-based Random walk with restart on heterogeneous network
PMF: Probabilistic matrix decomposition
PPI: Protein-protein interaction
SVT: Singular value thresholding
TDA: Topological data analysis

## References

[1] Nosengo N (2016). Can you teach old drugs new tricks?. Nature News.

[2] Alaimo S, Pulvirenti A (2019). Network-Based Drug Repositioning: Approaches, Resources, and Research Directions. Computational Methods for Drug Repurposing.

[3] van Laarhoven T, Marchiori E (2013). Predicting drug-target interactions for new drug compounds using a weighted nearest neighbor profile. PloS One.

[4] Mei J-P, Kwoh C-K, Yang P, Li X-L, Zheng J (2012). Drug–target interaction prediction by learning from local information and neighbors. Bioinformatics.

[5] Aliper A, Plis S, Artemov A, Ulloa A, Mamoshina P, Zhavoronkov A (2016). Deep learning applications for predicting pharmacological properties of drugs and drug repurposing using transcriptomic data. Mole Pharmaceutics.

[6] Xie L, Zhang Z, He S, Bo X, Song X (2017). Drug-target interaction prediction with a deep-learningbased model. Bioinformatics and Biomedicine (BIBM), 2017 IEEE International Conference On.

[7] Wen M, Zhang Z, Niu S, Sha H, Yang R, Yun Y, Lu H (2017). Deep-learning-based drug–target interaction prediction. J Proteome Res.

[8] Bahi M, Batouche M (2018). Drug-target interaction prediction in drug repositioning based on deep semisupervised learning. Computational Intelligence and Its Applications: 6th IFIP TC 5 International Conference, CIIA 2018, Oran, Algeria, May 8-10, 2018, Proceedings 6.

[9] Wang L, You Z-H, Chen X, Xia S-X, Liu F, Yan X, Zhou Y, Song K-J (2018). A computational-based method for predicting drug–target interactions by using stacked autoencoder deep neural network. J Comput Biol.

[10] Xuan P, Cao Y, Zhang T, Wang X, Pan S, Shen T (2019). Drug repositioning through integration of prior knowledge and projections of drugs and diseases. Bioinformatics.

[11] Zheng X, Ding H, Mamitsuka H, Zhu S (2013). Collaborative matrix factorization with multiple similarities for predicting drug-target interactions. Proceedings of the 19th ACM SIGKDD International Conference on Knowledge Discovery and Data Mining.

[12] Cobanoglu MC, Liu C, Hu F, Oltvai ZN, Bahar I (2013). Predicting drug–target interactions using probabilistic matrix factorization. J Chem Inf Model.

[13] Luo H, Li M, Wang S, Liu Q, Li Y, Wang J (2018). Computational drug repositioning using low-rank matrix approximation and randomized algorithms. Bioinformatics.

[14] Cheng F, Liu C, Jiang J, Lu W, Li W, Liu G, Zhou W, Huang J, Tang Y (2012). Prediction of drug-target interactions and drug repositioning via network-based inference. PLoS Comput Biol.

[15] Alaimo S, Pulvirenti A, Giugno R, Ferro A (2013). Drug–target interaction prediction through domain-tuned network-based inference. Bioinformatics.

[16] Chen X, Liu M-X, Yan G-Y (2012). Drug–target interaction prediction by random walk on the heterogeneous network. Mole BioSyst.

[17] Luo Y, Zhao X, Zhou J, Yang J, Zhang Y, Kuang W, Peng J, Chen L, Zeng J (2017). A network integration approach for drug-target interaction prediction and computational drug repositioning from heterogeneous information. Nature Commun.

[18] Lee T, Yoon Y (2018). Drug repositioning using drug-disease vectors based on an integrated network. BMC Bioinformatics.

[19] Tian Z, Teng Z, Cheng S, Guo M (2018). Computational drug repositioning using meta-path-based semantic network analysis. BMC Syst Biol.

[20] Kim I-W, Jang H, Kim JH, Kim MG, Kim S, Oh JM (2019). Computational drug repositioning for gastric cancer using reversal gene expression profiles. Sci Rep.

[21] Nagaraj A, Wang Q, Joseph P, Zheng C, Chen Y, Kovalenko O, Singh S, Armstrong A, Resnick K, Zanotti K (2018). Using a novel computational drug-repositioning approach (drugpredict) to rapidly identify potent drug candidates for cancer treatment. Oncogene.

[22] Xu C, Ai D, Suo S, Chen X, Yan Y, Cao Y, Sun N, Chen W, McDermott J, Zhang S (2018). Accurate drug repositioning through non-tissue-specific core signatures from cancer transcriptomes. Cell Rep.

[23] Zhou X, Dai E, Song Q, Ma X, Meng Q, Jiang Y, Jiang W (2019). In silico drug repositioning based on drug-mirna associations. Brief Bioinforma.

[24] Iwata M, Hirose L, Kohara H, Liao J, Sawada R, Akiyoshi S, Tani K, Yamanishi Y (2018). Pathway-based drug repositioning for cancers: Computational prediction and experimental validation. J Med Chem.

[25] Lee HS, Bae T, Lee J-H, Kim DG, Oh YS, Jang Y, Kim J-T, Lee J-J, Innocenti A, Supuran CT (2012). Rational drug repositioning guided by an integrated pharmacological network of protein, disease and drug. BMC Syst Biol.

[26] Singh G, Mémoli F, Carlsson GE (2007). Topological methods for the analysis of high dimensionaldata sets and 3d object recognition. Symposium on Point-Based Graphics.

[27] Lorente C, Arias JL, Cabeza L, Ortiz R, Prados JC, Melguizo C, Delgado ÁV, Clares-Naveros B (2018). Nano-engineering of biomedical prednisolone liposomes: evaluation of the cytotoxic effect on human colon carcinoma cell lines. J Phar Pharmacol.

[28] Patras L, Sylvester B, Luput L, Sesarman A, Licarete E, Porfire A, Muntean D, Drotar DM, Rusu AD, Nagy A-L (2017). Liposomal prednisolone phosphate potentiates the antitumor activity of liposomal 5-fluorouracil in c26 murine colon carcinoma in vivo. Canc Biol Ther.

[29] Wall I, Schmidt-Wolf IG (2014). Effect of wnt inhibitors in pancreatic cancer. Anticancer Res.

[30] Fujishita T, Kojima Y, Kajino-Sakamoto R, Taketo M, Aoki M (2017). Tumor microenvironment confers mtor inhibitor resistance in invasive intestinal adenocarcinoma. Oncogene.

[31] Wilton KM, Matteson EL (2017). Malignancy incidence, management, and prevention in patients with rheumatoid arthritis. Rheumatol Therapy.

[32] Alfaro-Lira S, Pizarro-Ortiz M, Calaf GM (2012). Malignant transformation of rat kidney induced by environmental substances and estrogen. Int J Environ Res Publ Health.

[33] Castiglioni S, Maier JA (2011). Magnesium and cancer: a dangerous liason. Magnesium Res.

[34] Pottegård A, Kristensen KB, Ernst MT, Johansen NB, Quartarolo P, Hallas J (2018). Use of n-nitrosodimethylamine (ndma) contaminated valsartan products and risk of cancer: Danish nationwide cohort study. bmj.

[35] Kanehisa M, Sato Y, Furumichi M, Morishima K, Tanabe M (2018). New approach for understanding genome variations in kegg. Nucleic Acids Res.

[36] Wazea SA, Wadie W, Bahgat AK, El-Abhar HS (2018). Galantamine anti-colitic effect: Role of alpha-7 nicotinic acetylcholine receptor in modulating jak/stat3, nf- *κ*b/hmgb1/rage and p-akt/bcl-2 pathways. Sci Rep.

[37] Zhang C, Li S, Sun Y, Dong W, Piao F, Piao Y, Guan H, Yu S (2014). Arsenic downregulates gene expression at the postsynaptic density in mouse cerebellum, including genes responsible for long-term potentiation and depression. Toxicol Lett.

[38] Knox C, Law V, Jewison T, Liu P, Ly S, Frolkis A, Pon A, Banco K, Mak C, Neveu V (2010). Drugbank 3.0: a comprehensive resource for ’omics’ research on drugs. Nucleic Acids Res.

[39] Keshava Prasad T, Goel R, Kandasamy K, Keerthikumar S, Kumar S, Mathivanan S, Telikicherla D, Raju R, Shafreen B, Venugopal A (2008). Human protein reference database—2009 update. Nucleic acids Res.

[40] Davis AP, Murphy CG, Johnson R, Lay JM, Lennon-Hopkins K, Saraceni-Richards C, Sciaky D, King BL, Rosenstein MC, Wiegers TC (2012). The comparative toxicogenomics database: update 2013. Nucleic Acids Res.

[41] Wang R, Li S, Wong MH, Leung KS (2018). Drug-protein-disease association prediction and drug repositioning based on tensor decomposition. Bioinformatics and Biomedicine (BIBM), 2018 IEEE International Conference On.

[42] Sawada R, Iwata H, Mizutani S, Yamanishi Y (2015). Target-based drug repositioning using large-scale chemical–protein interactome data. J Chem Inf Model.

[43] Ashburner M, Ball CA, Blake JA, Botstein D, Butler H, Cherry JM, Davis AP, Dolinski K, Dwight SS, Eppig JT (2000). Gene ontology: tool for the unification of biology. Nature genetics.

[44] Consortium GO (2018). The gene ontology resource: 20 years and still going strong. Nucleic Acids Res.

[45] Tan K, Tesar C, Wilton R, Jedrzejczak RP, Joachimiak A (2018). The interaction of anti-diabetic *α*-glucosidase inhibitors and gut bacteria *α*-glucosidase. Protein Sci.

[46] Eberhart K, Oral O, Gozuacik D (2014). Induction of autophagic cell death by anticancer agents. Autophagy: Cancer, Other Pathologies, Inflammation, Immunity, Infection, and Aging.

[47] Shihab F, Christians U, Smith L, Wellen JR, Kaplan B (2014). Focus on mtor inhibitors and tacrolimus in renal transplantation: Pharmacokinetics, exposure–response relationships, and clinical outcomes. Trans Immun.

