# Supplementary material for: Predicting associations among drugs, targets and diseases by tensor decomposition for drug repositioning
Source: BMC Bioinformatics. 2019 Dec 16;20(Suppl 26):628. doi: 10.1186/s12859-019-3283-6 (PMC6912989; doi:10.1186/s12859-019-3283-6)
Supplement: Supplementary file 1 — Additional file 1 Figure S1. Performance of decomposing χtri with different additional information. [file 12859_2019_3283_MOESM1_ESM.pdf]

A

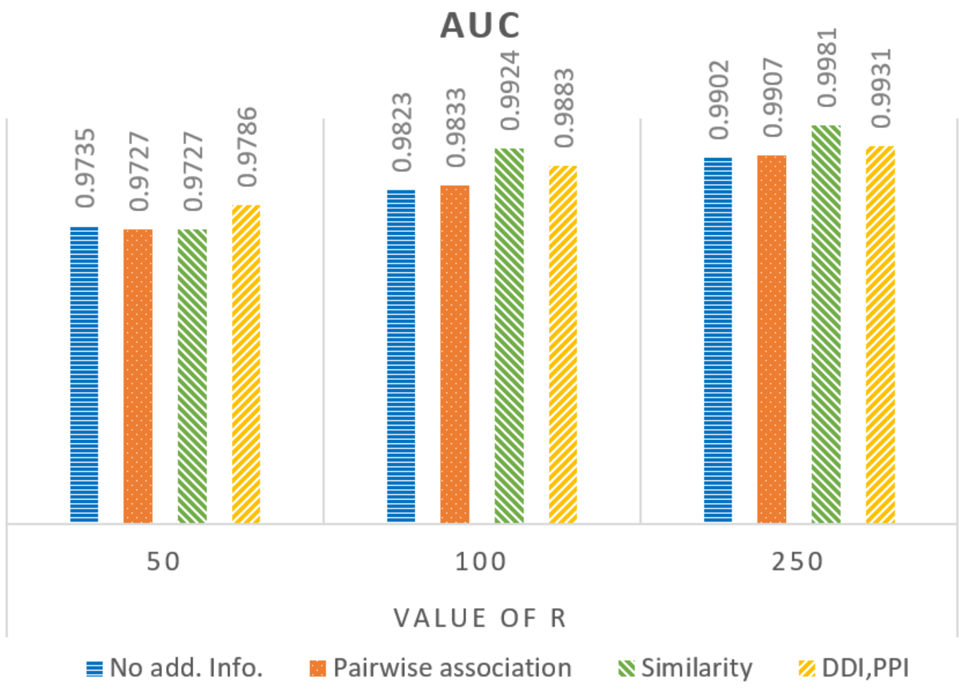

B

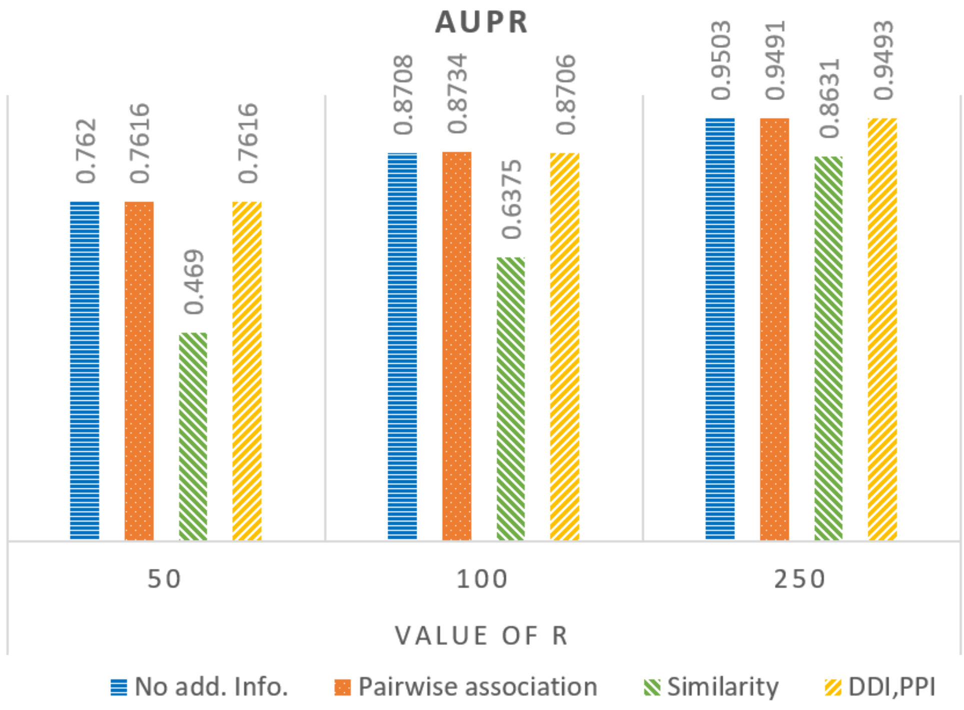

Figure S1. Performance of decomposing χ^{tri} with different additional information. AUC (**a**) and AUPR (**b**) evaluated under different number of latent factors (R) are illustrated. No add. Info., using no additional information.
