# Supplementary material for: Predicting associations among drugs, targets and diseases by tensor decomposition for drug repositioning
Source: BMC Bioinformatics. 2019 Dec 16;20(Suppl 26):628. doi: 10.1186/s12859-019-3283-6 (PMC6912989; doi:10.1186/s12859-019-3283-6)
Supplement: Supplementary file 2 — Additional file 2 Figure S2. Performance of decomposing the five random tensors constructed by the second strategy. [file 12859_2019_3283_MOESM2_ESM.pdf]

A

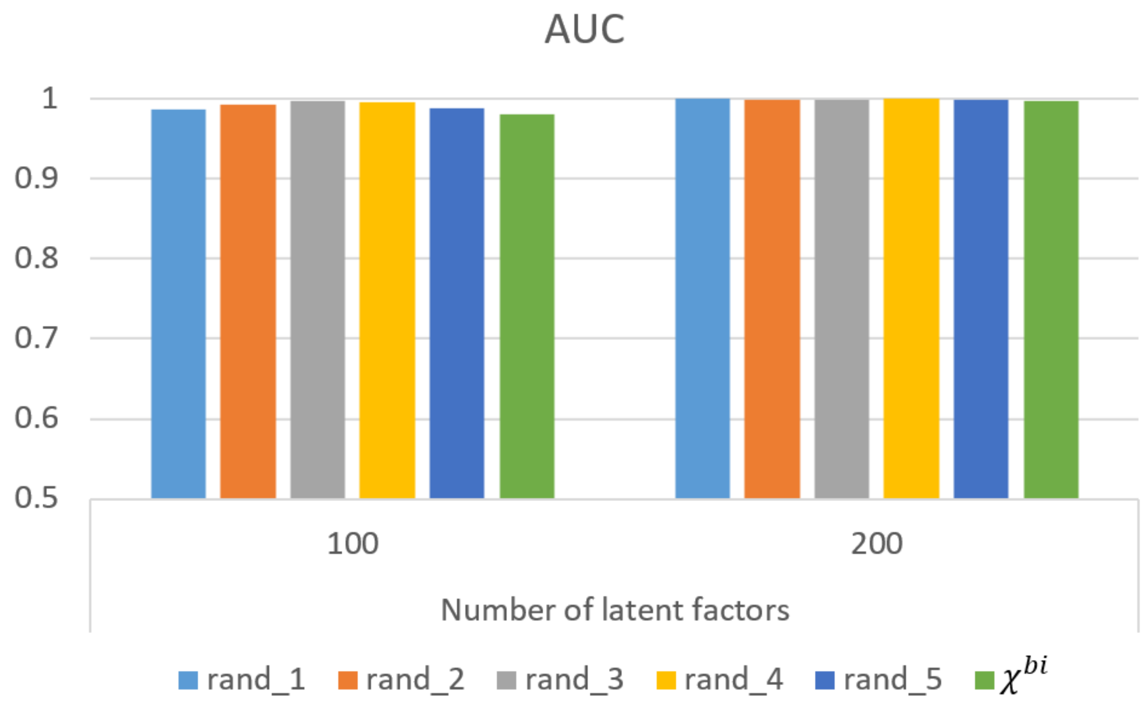
AUC
1
0.9
0.8
0.7
0.6
0.5
100
200
Number of latent factors
rand_1
rand_2
rand_3
rand_4
rand_5
χ^{bi}

B

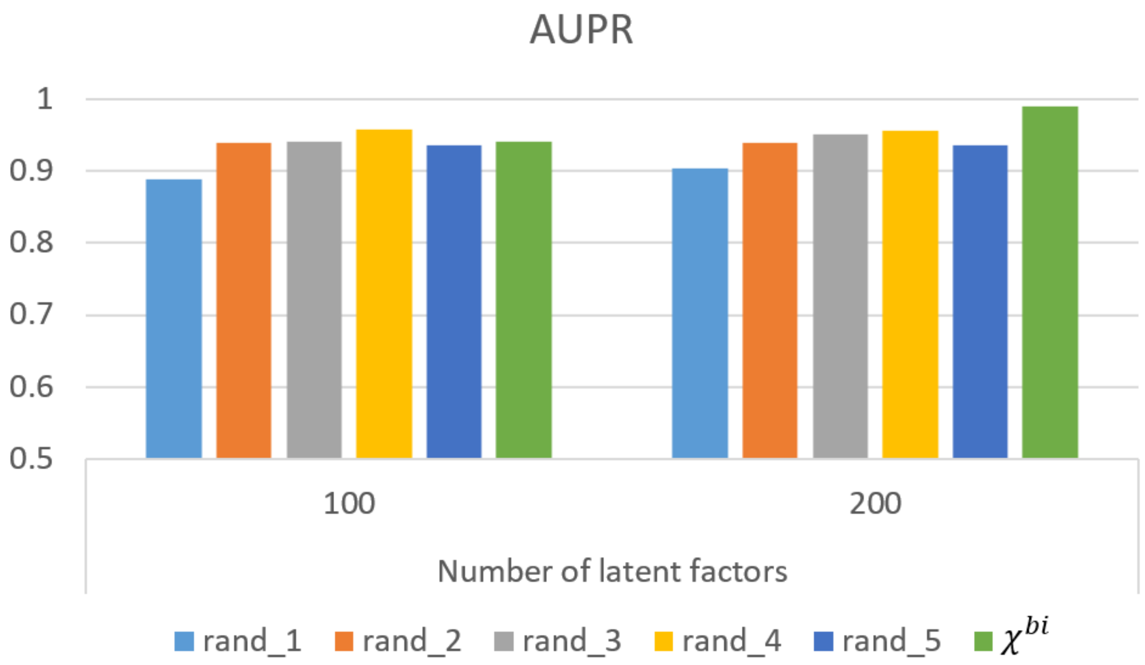
AUPR
1
0.9
0.8
0.7
0.6
0.5
100
200
Number of latent factors
rand_1
rand_2
rand_3
rand_4
rand_5
χ^{bi}

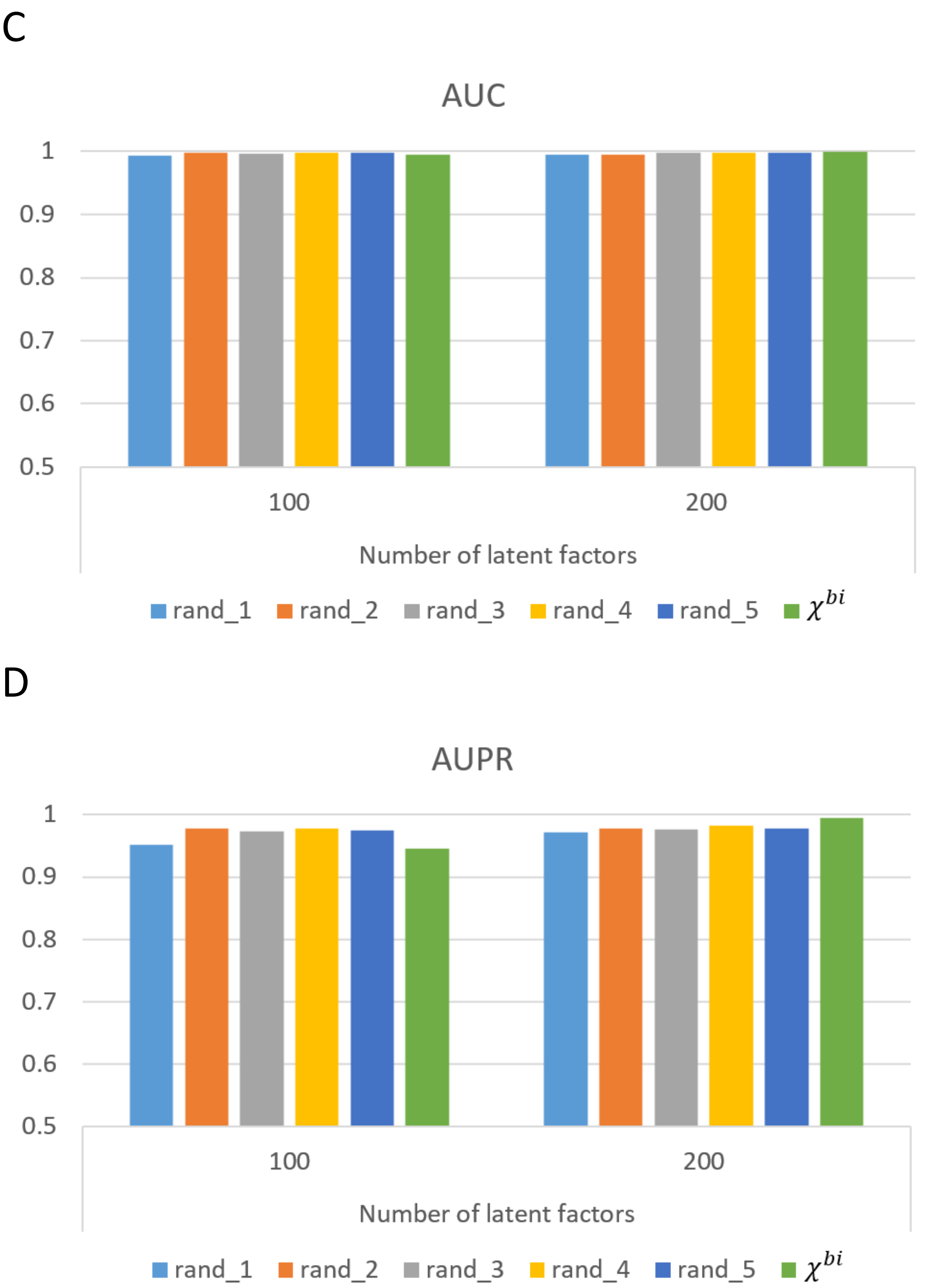

Figure S2. Performance of decomposing the five random tensors constructed by the second strategy. Performance of using different number of latent factors is demonstrated, compared with performance of decomposing χ^{bi}. **a** and **b**, AUC and AUPR with no additional information, respectively. **c** and **d**, AUC and AUPR using similarity as additional information, respectively.
