# Supplementary material for: Predicting associations among drugs, targets and diseases by tensor decomposition for drug repositioning
Source: BMC Bioinformatics. 2019 Dec 16;20(Suppl 26):628. doi: 10.1186/s12859-019-3283-6 (PMC6912989; doi:10.1186/s12859-019-3283-6)
Supplement: Supplementary file 3 — Additional file 3 Figure S3. Boxplot of association enrichment in random tensors constructed by the second strategy. [file 12859_2019_3283_MOESM3_ESM.pdf]

A

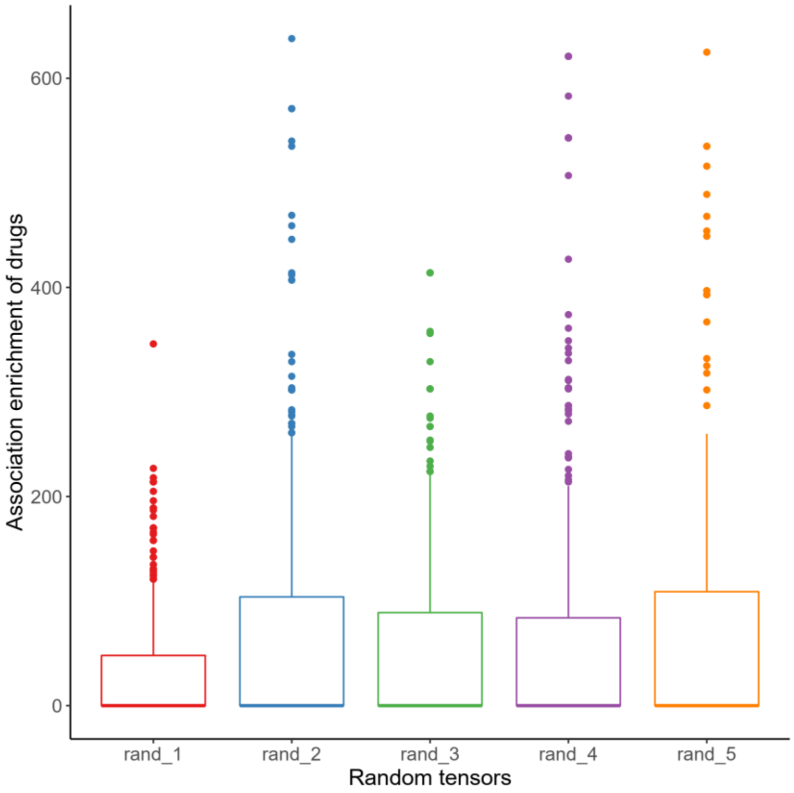

Association enrichment of drugs
0
200
400
600
rand_1
rand_2
rand_3
rand_4
rand_5
Random tensors

B

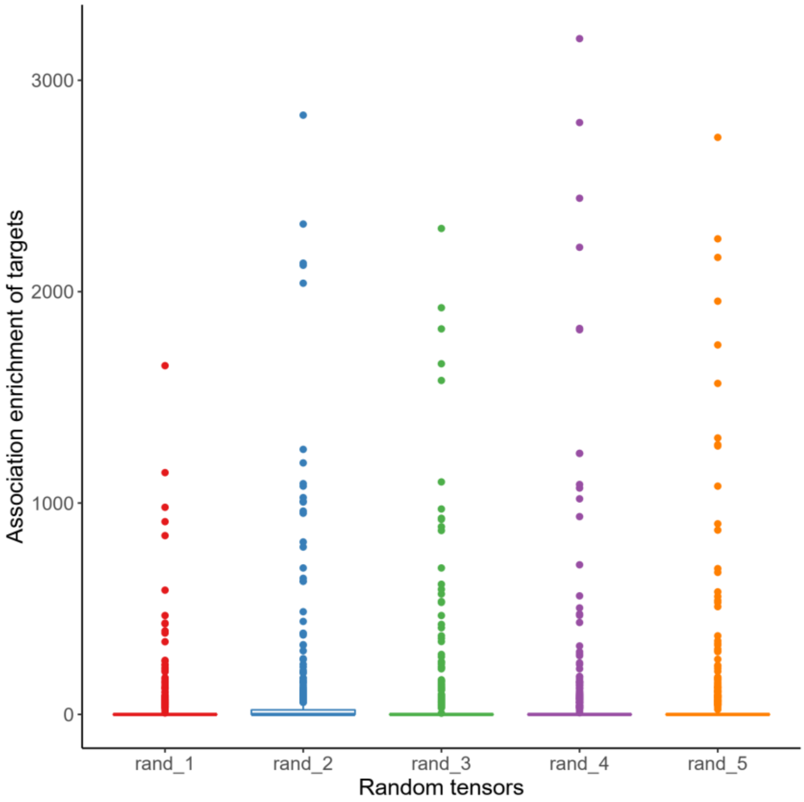

Association enrichment of targets
0
1000
2000
3000
rand_1
rand_2
rand_3
rand_4
rand_5
Random tensors

C

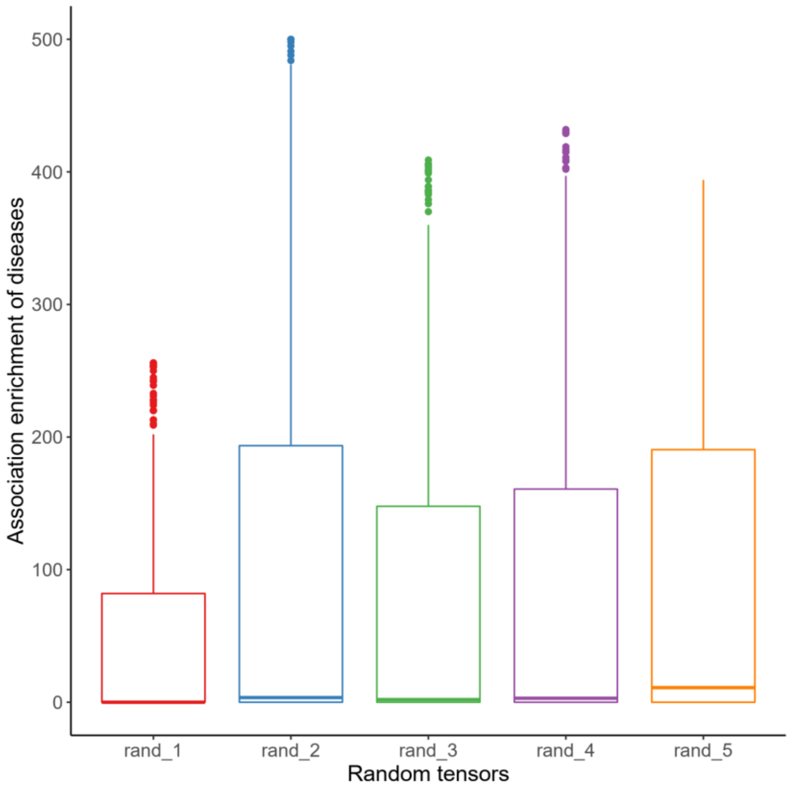

Figure S3. Boxplot of association enrichment in random tensors constructed by the second strategy. Association enrichment of drugs (**a**), targets (**b**) and diseases (**c**).
