# Supplementary material for: Predicting associations among drugs, targets and diseases by tensor decomposition for drug repositioning
Source: BMC Bioinformatics. 2019 Dec 16;20(Suppl 26):628. doi: 10.1186/s12859-019-3283-6 (PMC6912989; doi:10.1186/s12859-019-3283-6)
Supplement: Supplementary file 4 — Additional file 4 Figure S4. Similarity of triplet associaiton patterns of drug pairs (a), target pairs (b) and disease pairs (c) in the five random tensors constructed by the second strategy. [file 12859_2019_3283_MOESM4_ESM.pdf]

A

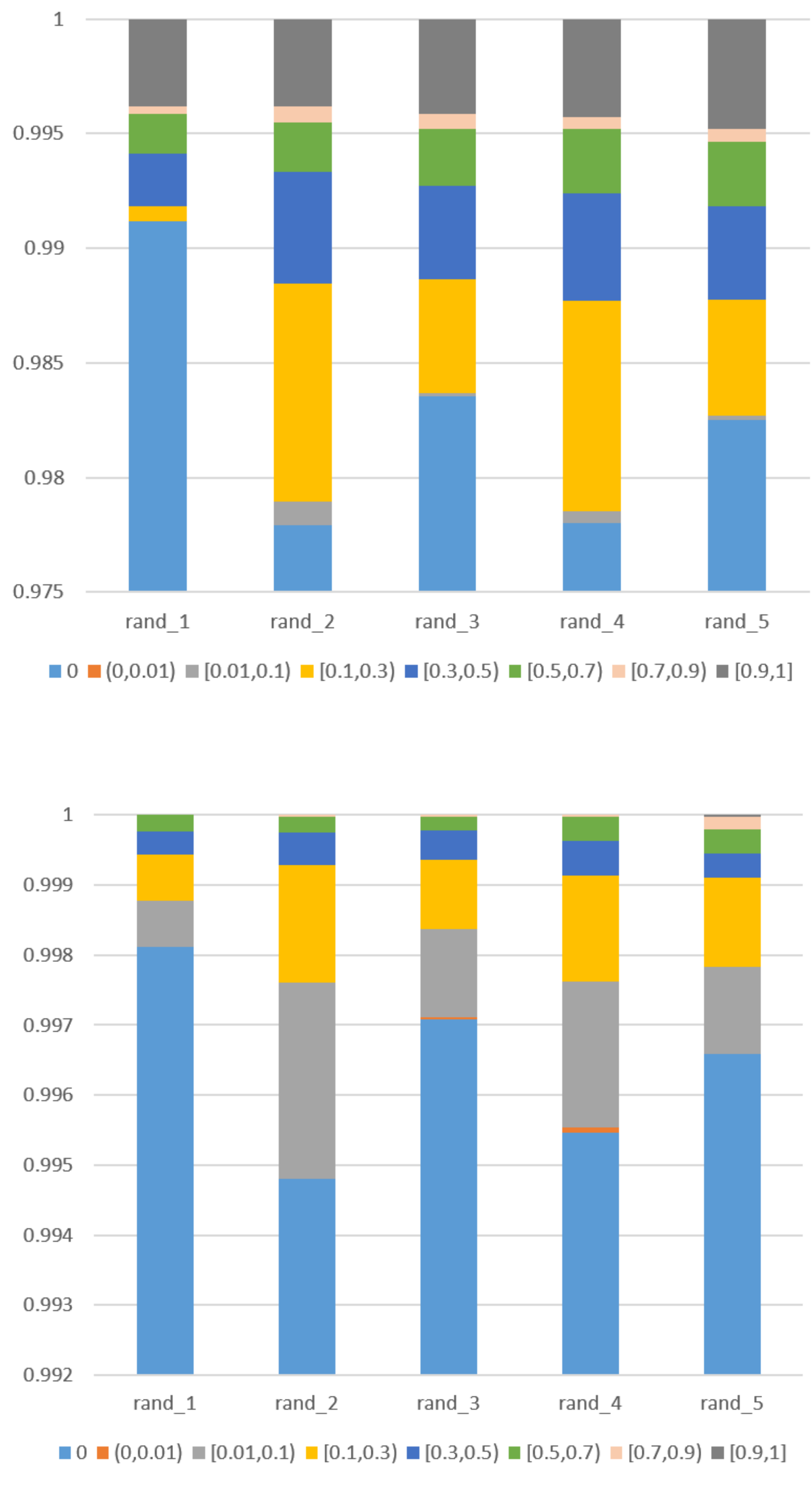

1
0.995
0.99
0.985
0.98
0.975
rand_1
rand_2
rand_3
rand_4
rand_5
0
(0,0.01)
[0.01,0.1)
[0.1,0.3)
[0.3,0.5)
[0.5,0.7)
[0.7,0.9)
[0.9,1]
B
1
0.999
0.998
0.997
0.996
0.995
0.994
0.993
0.992
rand_1
rand_2
rand_3
rand_4
rand_5
0
(0,0.01)
[0.01,0.1)
[0.1,0.3)
[0.3,0.5)
[0.5,0.7)
[0.7,0.9)
[0.9,1]

C

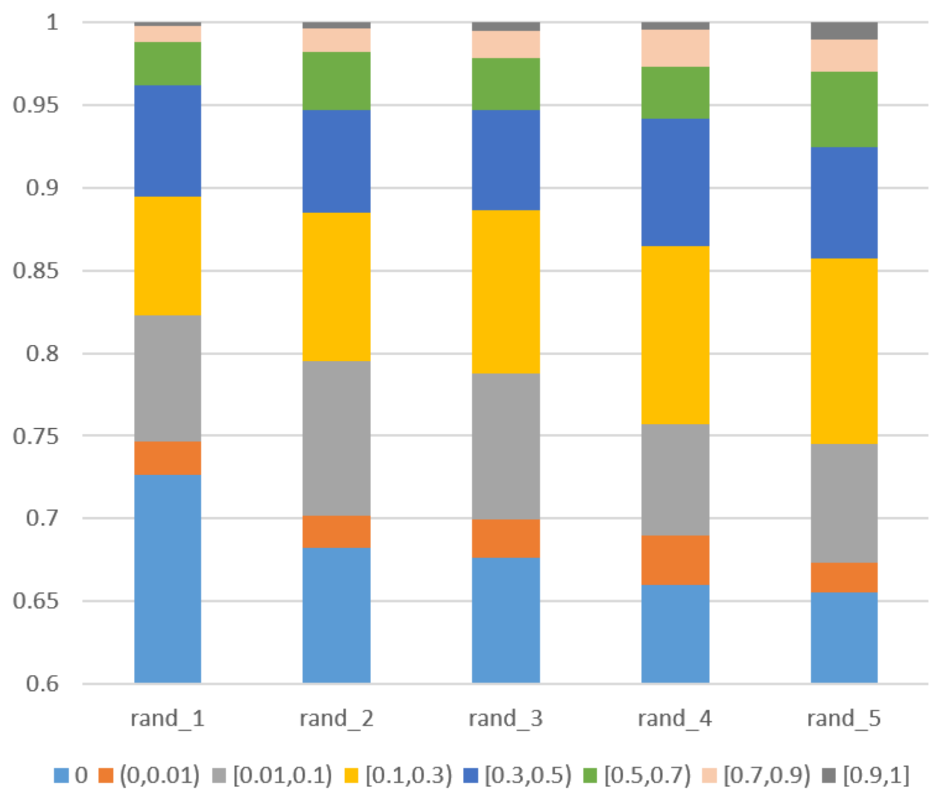

Figure S4. Similarity of triplet associaiton patterns of drug pairs (a), target pairs (b) and disease pairs (c) in the five random tensors constructed by the second strategy. X-axis represents different random tensors. Y-axis represents the cumulative proportion of different ranges of Jaccard similarity. Different colors demonstrate different ranges of similarity.
