# Supplementary material for: Predicting associations among drugs, targets and diseases by tensor decomposition for drug repositioning
Source: BMC Bioinformatics. 2019 Dec 16;20(Suppl 26):628. doi: 10.1186/s12859-019-3283-6 (PMC6912989; doi:10.1186/s12859-019-3283-6)
Supplement: Supplementary file 5 — Additional file 5 Figure S5. Boxplot of similarity of triplet associaiton patterns of drug pairs (a), target pairs (b) and disease pairs (c) in the five random tensors constructed by the first strategy. [file 12859_2019_3283_MOESM5_ESM.pdf]

A

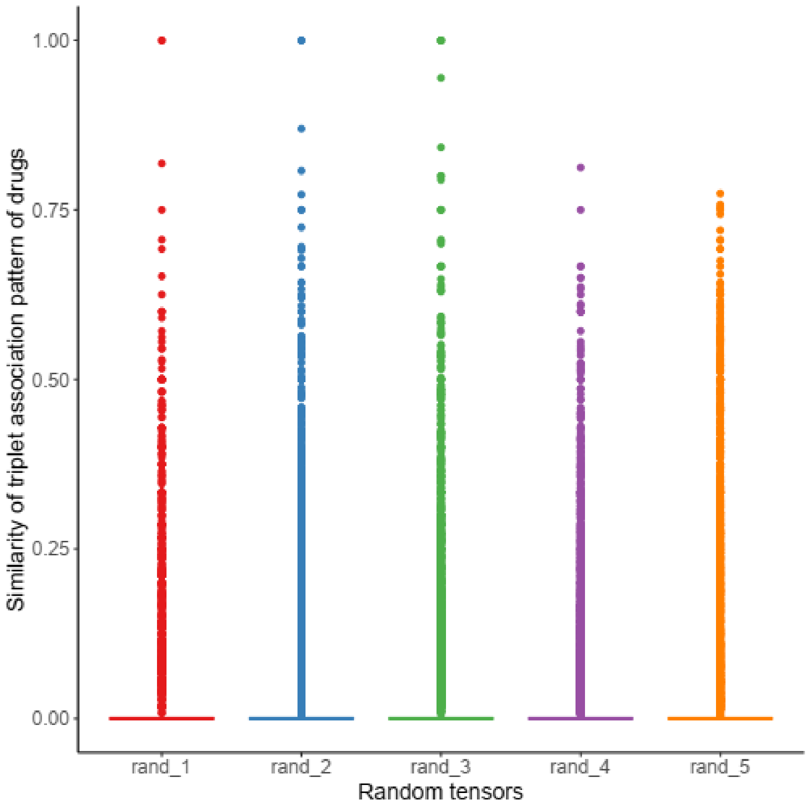
Similarity of triplet association pattern of drugs
1.00
0.75
0.50
0.25
0.00
rand_1
rand_2
rand_3
rand_4
rand_5
Random tensors

B

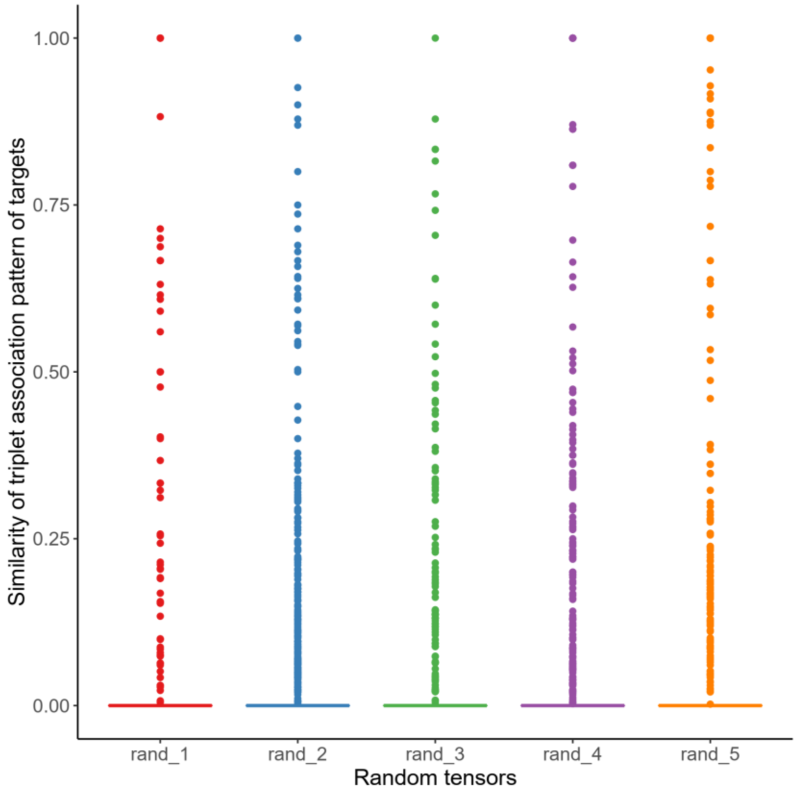
Similarity of triplet association pattern of targets
1.00
0.75
0.50
0.25
0.00
rand_1
rand_2
rand_3
rand_4
rand_5
Random tensors

C

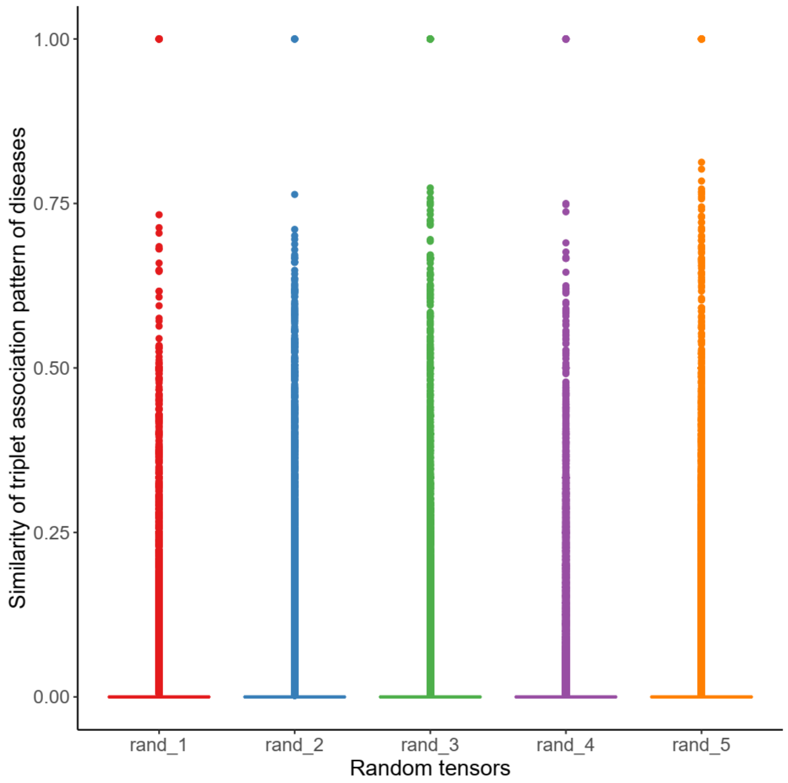

Figure S5. Boxplot of similarity of triplet associaiton patterns of drug pairs (a), target pairs (b) and disease pairs (c) in the five random tensors constructed by the first strategy.
