# Supplementary material for: Predicting associations among drugs, targets and diseases by tensor decomposition for drug repositioning
Source: BMC Bioinformatics. 2019 Dec 16;20(Suppl 26):628. doi: 10.1186/s12859-019-3283-6 (PMC6912989; doi:10.1186/s12859-019-3283-6)
Supplement: Supplementary file 7 — Additional file 7 Figure S7. Boxplot of association enrichment in random tensors constructed by the second strategy, compared to that of χbi. [file 12859_2019_3283_MOESM7_ESM.pdf]

A

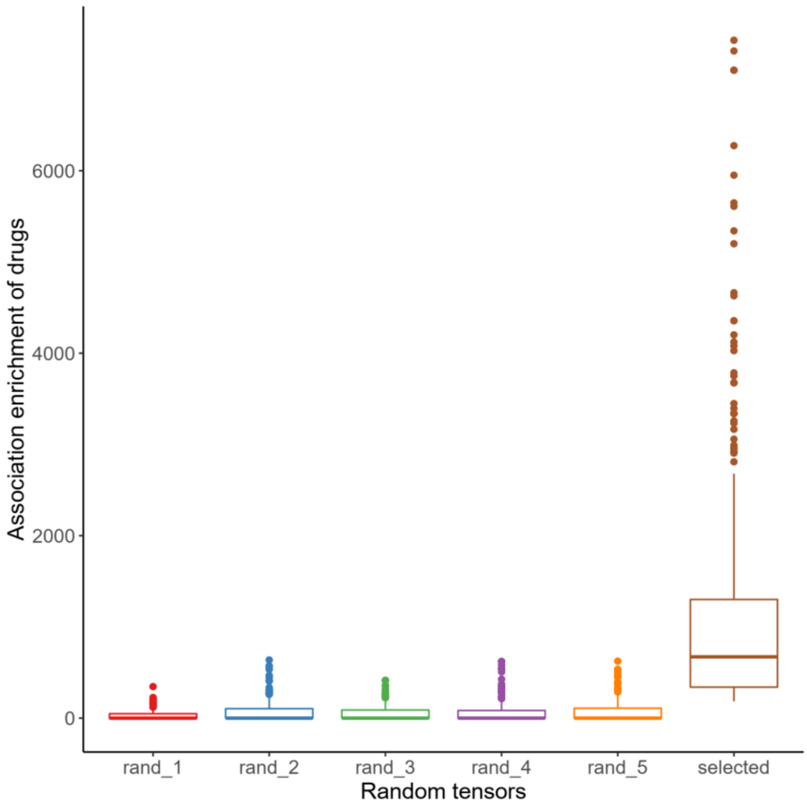
Association enrichment of drugs
6000
4000
2000
0
rand_1
rand_2
rand_3
rand_4
rand_5
selected
Random tensors

B

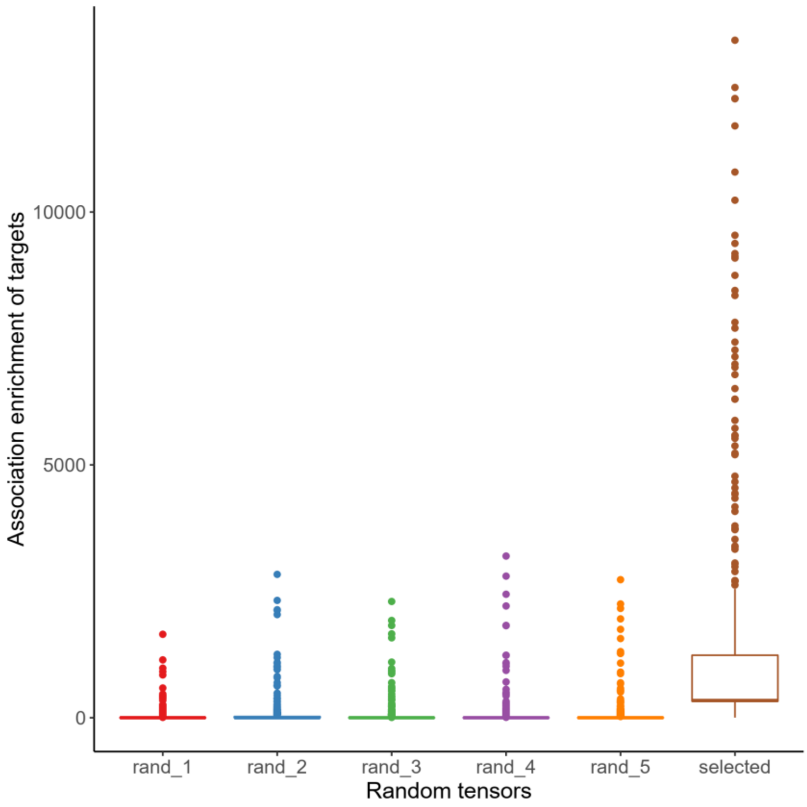
Association enrichment of targets
10000
5000
0
rand_1
rand_2
rand_3
rand_4
rand_5
selected
Random tensors

c

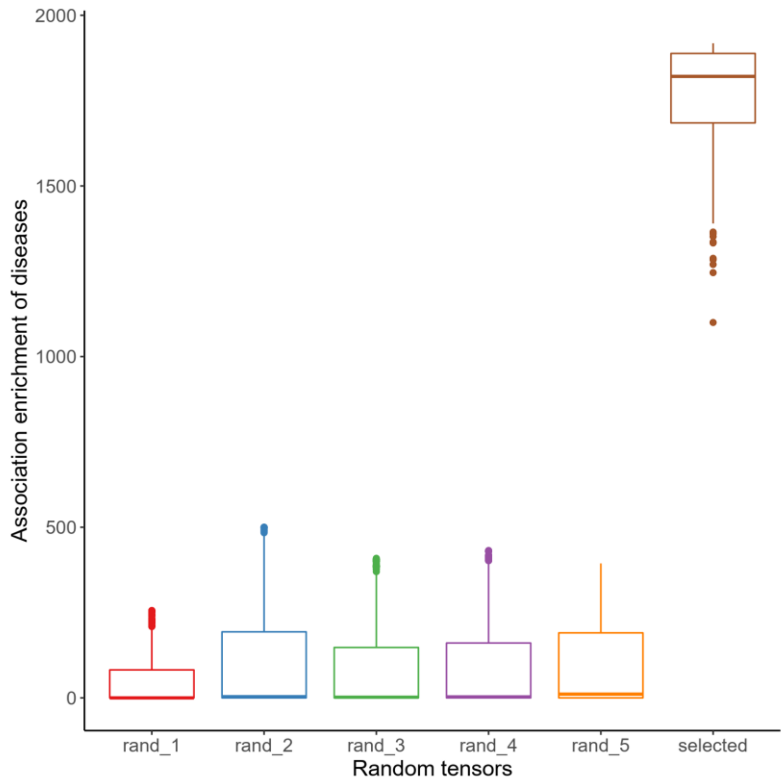

Figure S7. Boxplot of association enrichment in random tensors constructed by the second strategy, compared to that of χ^{bi}. Association enrichment of drugs (**a**), targets (**b**) and diseases (**c**).
