# Supplementary material for: Predicting associations among drugs, targets and diseases by tensor decomposition for drug repositioning
Source: BMC Bioinformatics. 2019 Dec 16;20(Suppl 26):628. doi: 10.1186/s12859-019-3283-6 (PMC6912989; doi:10.1186/s12859-019-3283-6)
Supplement: Supplementary file 8 — Additional file 8 Figure S8. Boxplot of association enrichment in random tensors constructed by the first strategy, compared to that of χtri. [file 12859_2019_3283_MOESM8_ESM.pdf]

A

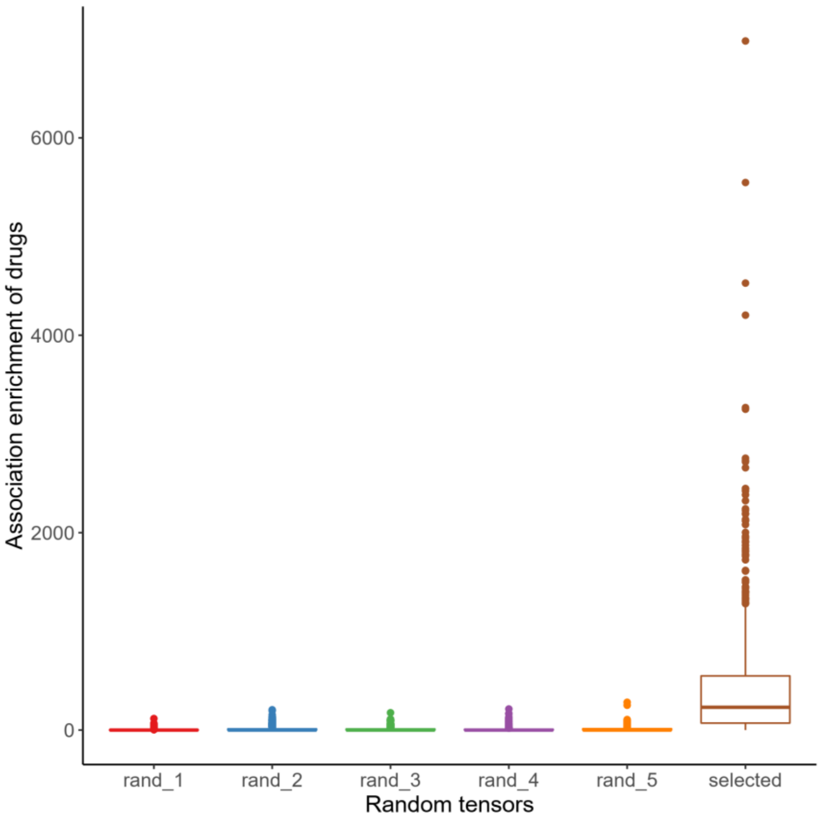

6000
4000
2000
0
Association enrichment of drugs
rand_1
rand_2
rand_3
rand_4
rand_5
selected
Random tensors

B

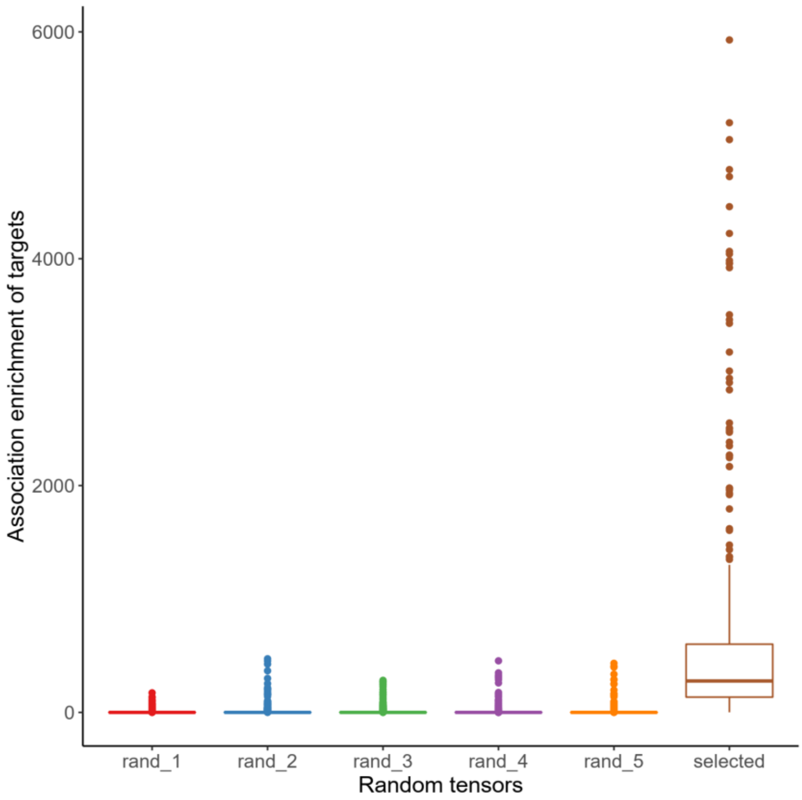

6000
4000
2000
0
Association enrichment of targets
rand_1
rand_2
rand_3
rand_4
rand_5
selected
Random tensors

c

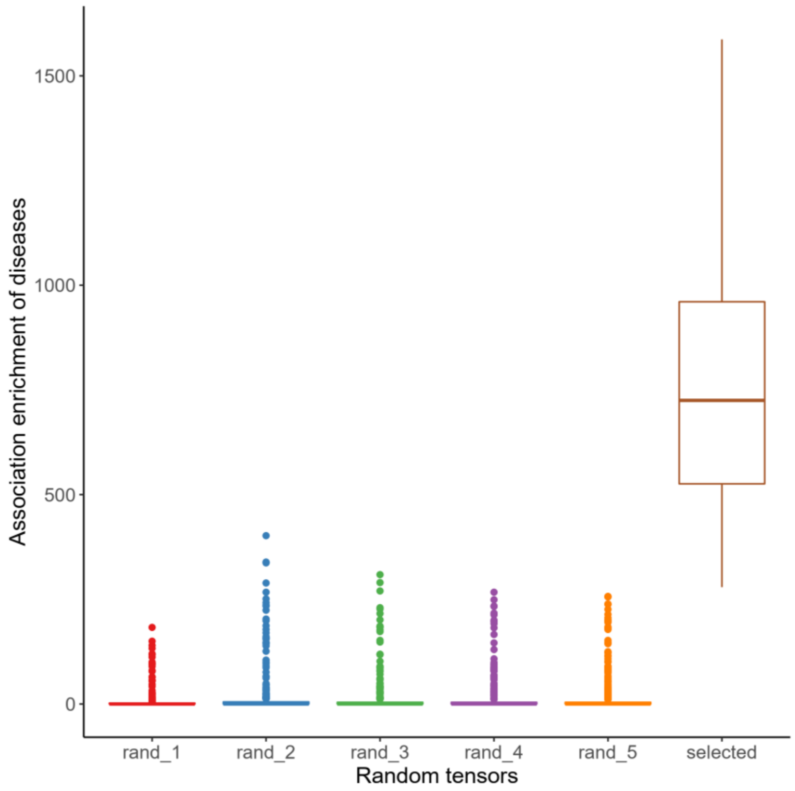

Figure S8. Boxplot of association enrichment in random tensors constructed by the first strategy, compared to that of χ^{tri}. Association enrichment of drugs (**a**), targets (**b**) and diseases (**c**).
