# Supplementary material for: Predicting associations among drugs, targets and diseases by tensor decomposition for drug repositioning
Source: BMC Bioinformatics. 2019 Dec 16;20(Suppl 26):628. doi: 10.1186/s12859-019-3283-6 (PMC6912989; doi:10.1186/s12859-019-3283-6)
Supplement: Supplementary file 9 — Additional file 9 Figure S9. Illustration of the baseline methods. [file 12859_2019_3283_MOESM9_ESM.pdf]

A

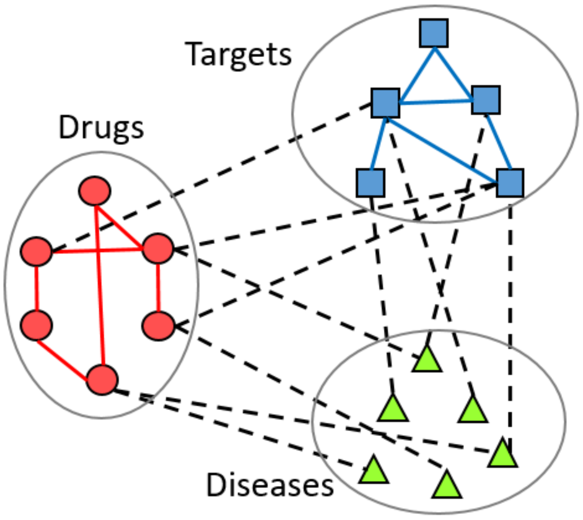

B

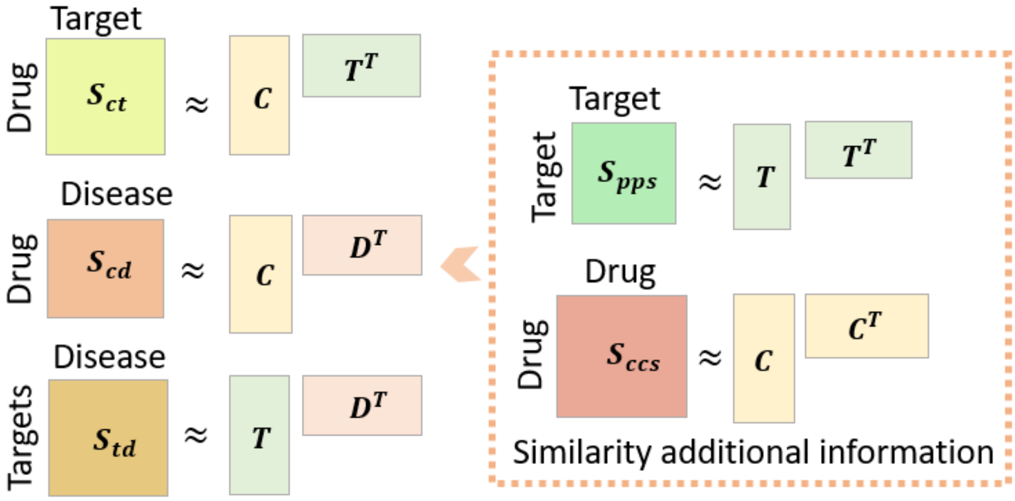

Figure S9. Illustration of the baseline methods. a Example heterogeneous network constructed for Network-based Random Walk with Restart on Heterogeneous network (NRWRH). **b** Demonstration of Collective Matrix Factorization (CMF).
