# Supplementary material for: Predicting associations among drugs, targets and diseases by tensor decomposition for drug repositioning
Source: BMC Bioinformatics. 2019 Dec 16;20(Suppl 26):628. doi: 10.1186/s12859-019-3283-6 (PMC6912989; doi:10.1186/s12859-019-3283-6)
Supplement: Supplementary file 10 — Additional file 10 Figure S10. Distribution of retrieved inference scores in CTD. [file 12859_2019_3283_MOESM10_ESM.pdf]

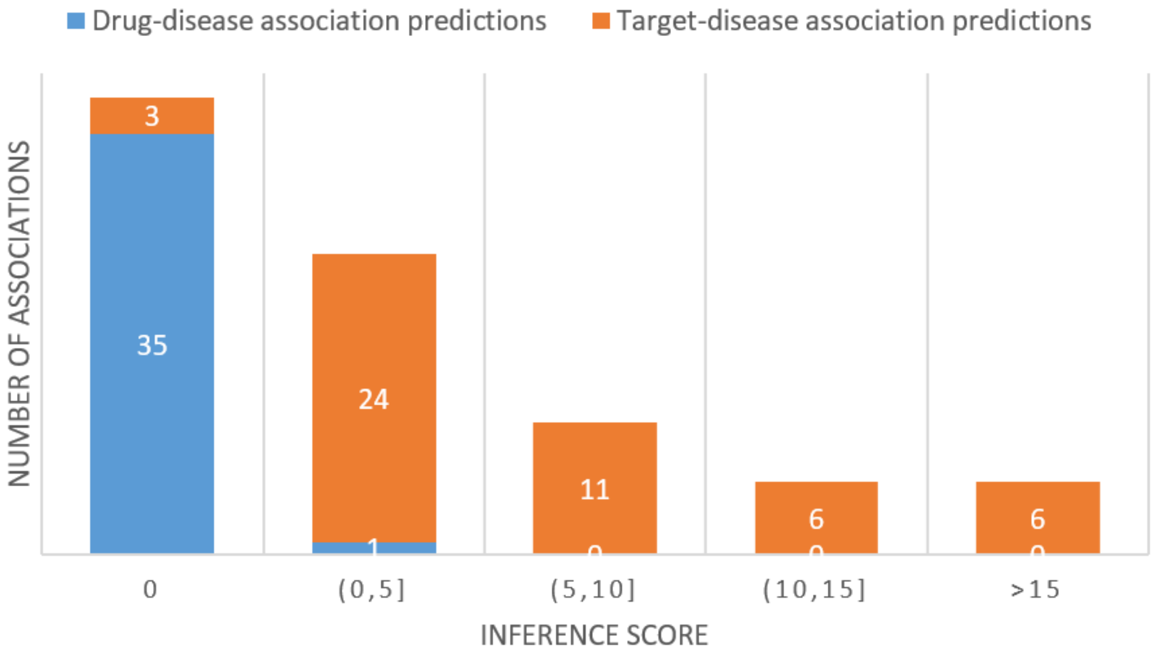

Figure S10. Distribution of retrieved inference scores in CTD. The distribution of inference scores of the drug-disease associations and target-disease associations are illustrated in different colors.
