# Supplementary material for: Predicting associations among drugs, targets and diseases by tensor decomposition for drug repositioning
Source: BMC Bioinformatics. 2019 Dec 16;20(Suppl 26):628. doi: 10.1186/s12859-019-3283-6 (PMC6912989; doi:10.1186/s12859-019-3283-6)
Supplement: Supplementary file 11 — Additional file 11 Figure S11. Docking poses for three top-ranking drug-target predictions. [file 12859_2019_3283_MOESM11_ESM.pdf]

A

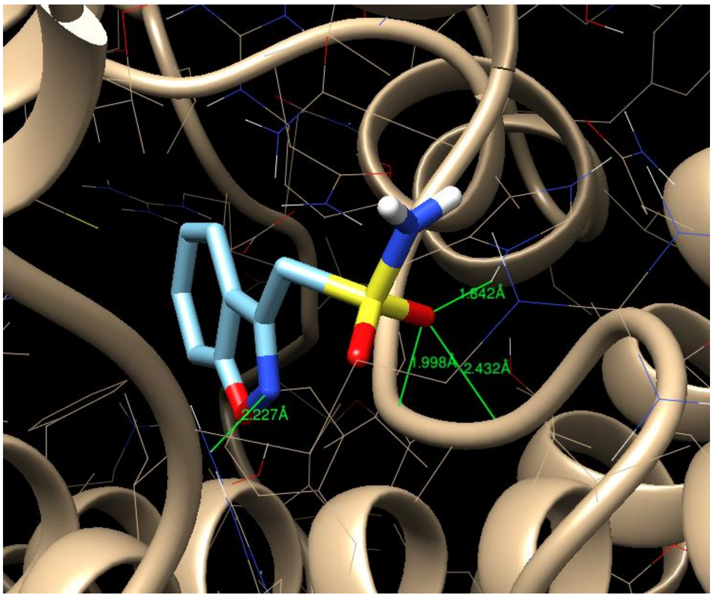

B

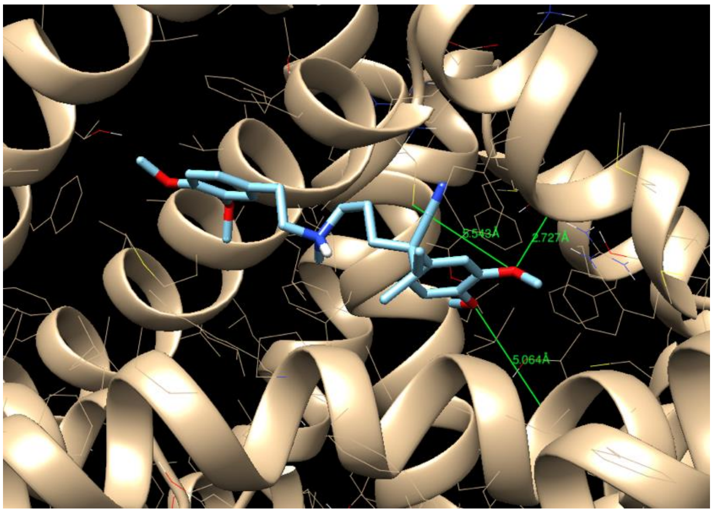

C

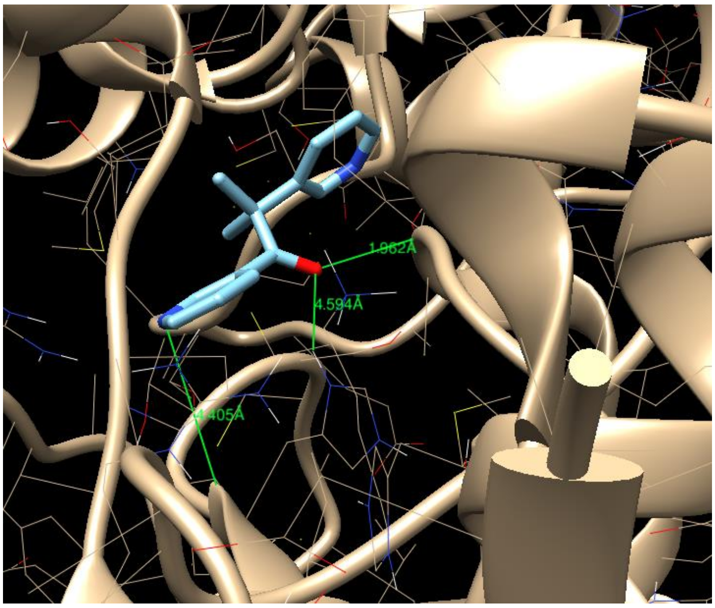

Figure S11. Docking poses for three top-ranking drug-target predictions. a Zonisamide vs. AQP1. **b** Verapamil vs. KCNK1. **c** Metyrapone vs. FDX1.
