# Supplementary material for: Predicting associations among drugs, targets and diseases by tensor decomposition for drug repositioning
Source: BMC Bioinformatics. 2019 Dec 16;20(Suppl 26):628. doi: 10.1186/s12859-019-3283-6 (PMC6912989; doi:10.1186/s12859-019-3283-6)
Supplement: Supplementary file 12 — Additional file 12 Figure S12. Correlation of latent factors derived from χbi. [file 12859_2019_3283_MOESM12_ESM.pdf]

A

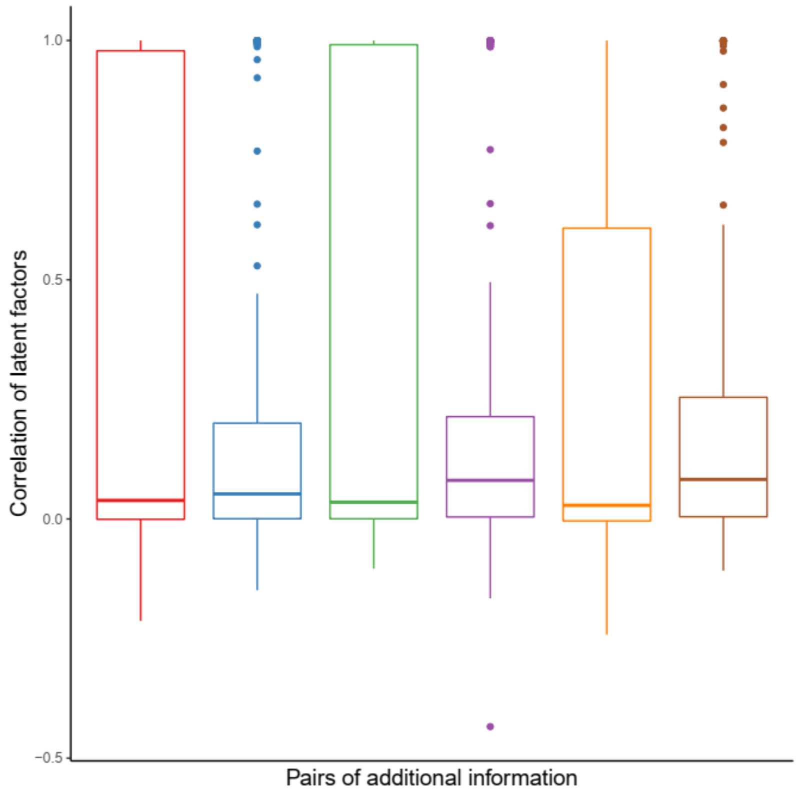
1.0
0.5
0.0
−0.5
Correlation of latent factors
Pairs of additional information

B

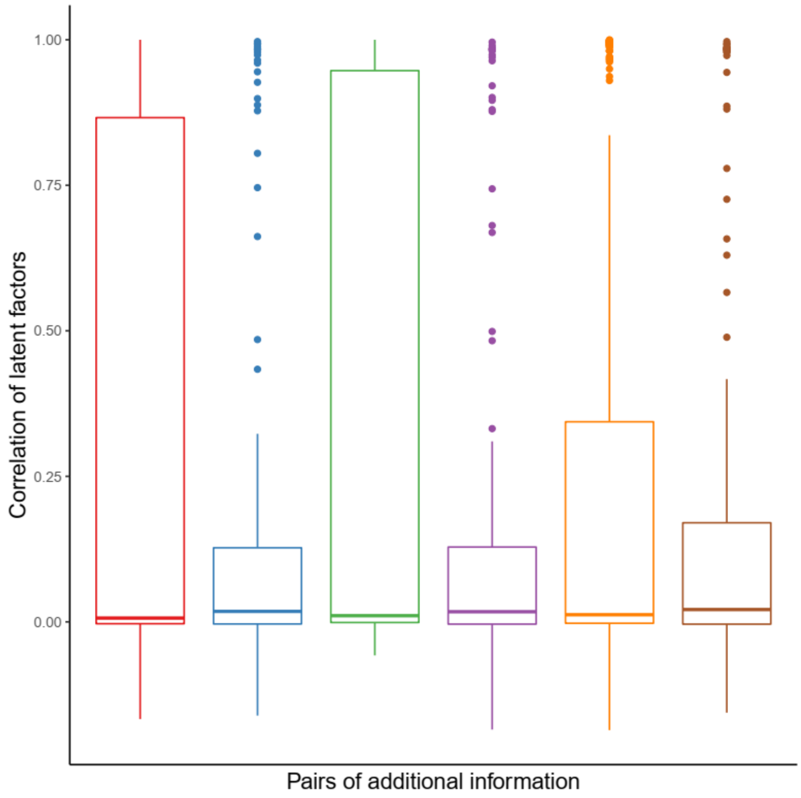
1.00
0.75
0.50
0.25
0.00
Correlation of latent factors
Pairs of additional information

c

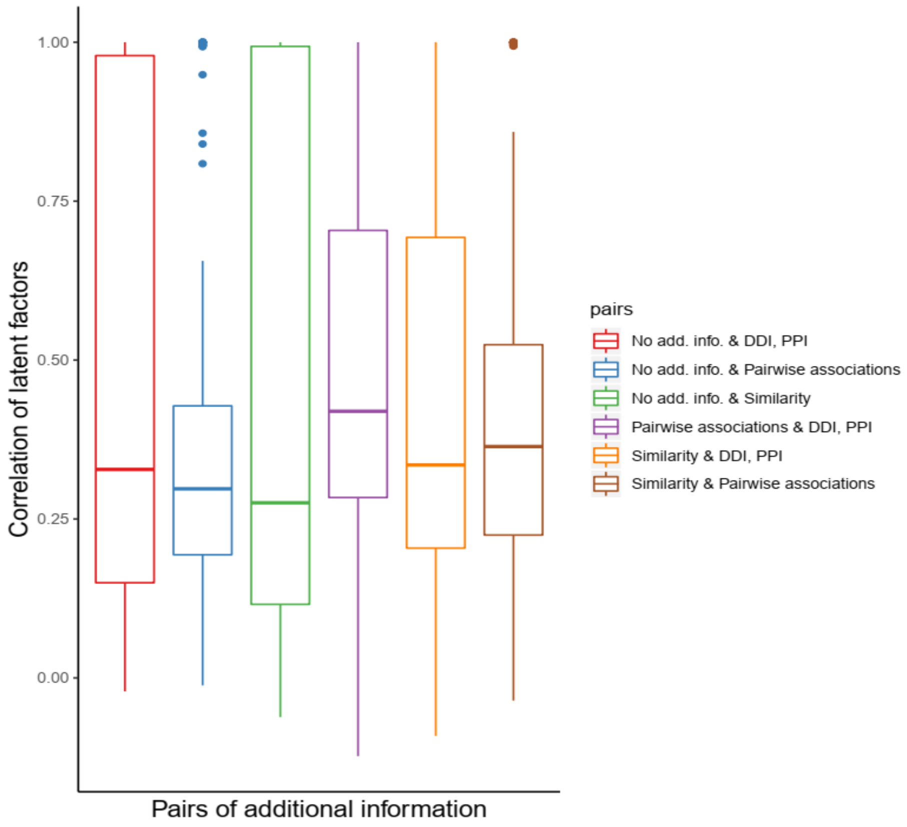

Figure S12. Correlation of latent factors derived from χ^{bi}. Different kinds of additional information are used. **a** Latent factors of drugs. **b** Latent factors of targets. **c** Latent factors of diseases. No add. Info., using no additional information.
