# Supplementary material for: Predicting associations among drugs, targets and diseases by tensor decomposition for drug repositioning
Source: BMC Bioinformatics. 2019 Dec 16;20(Suppl 26):628. doi: 10.1186/s12859-019-3283-6 (PMC6912989; doi:10.1186/s12859-019-3283-6)
Supplement: Supplementary file 13 — Additional file 13 Figure S13. Topological data analysis of drugs in terms of drug classes. [file 12859_2019_3283_MOESM13_ESM.pdf]

A

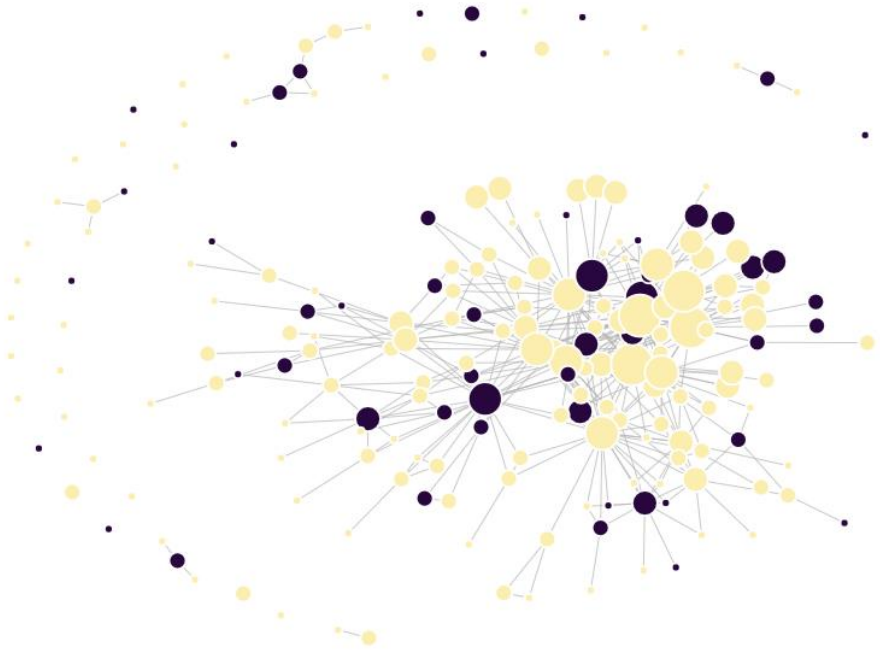

B

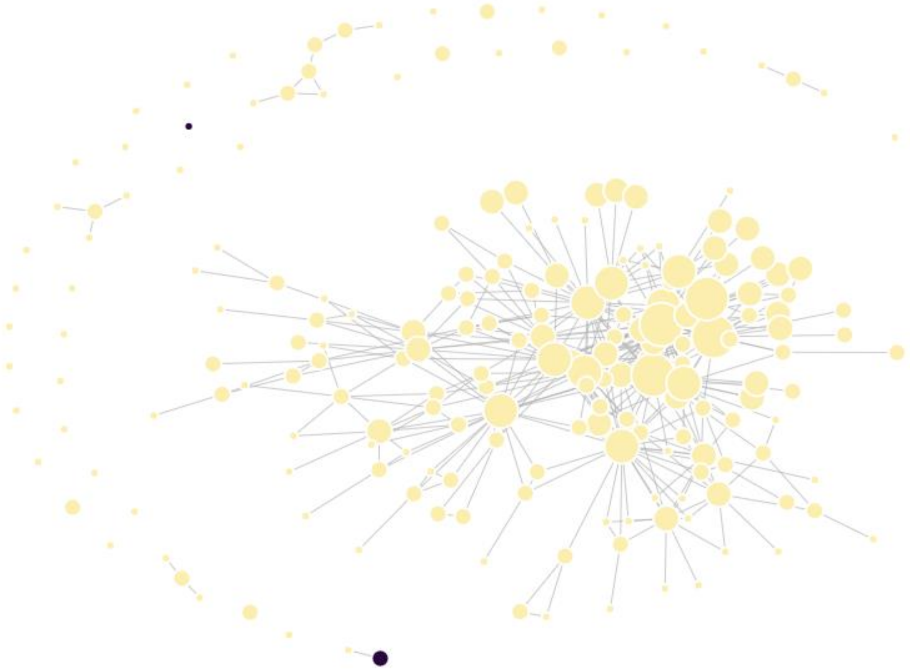

C

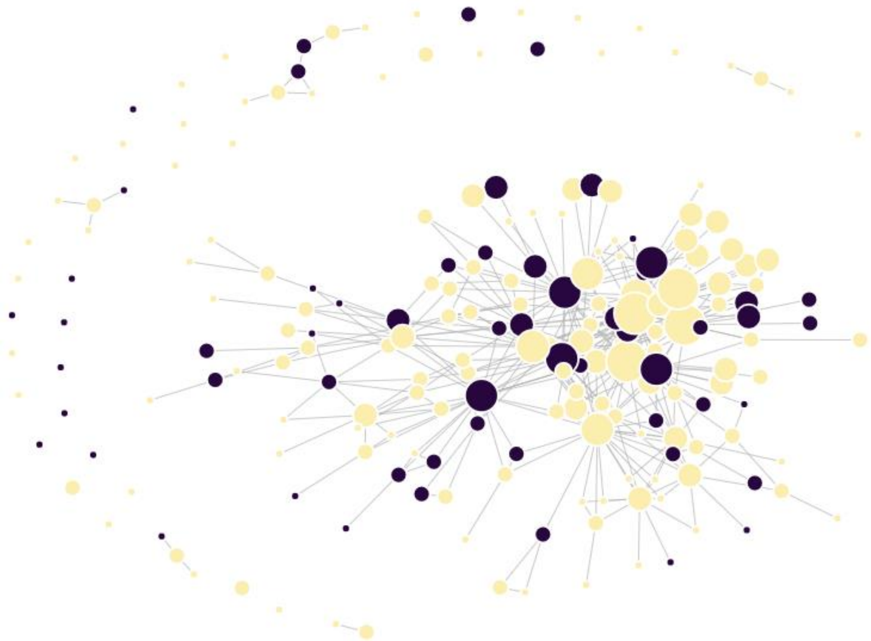

D

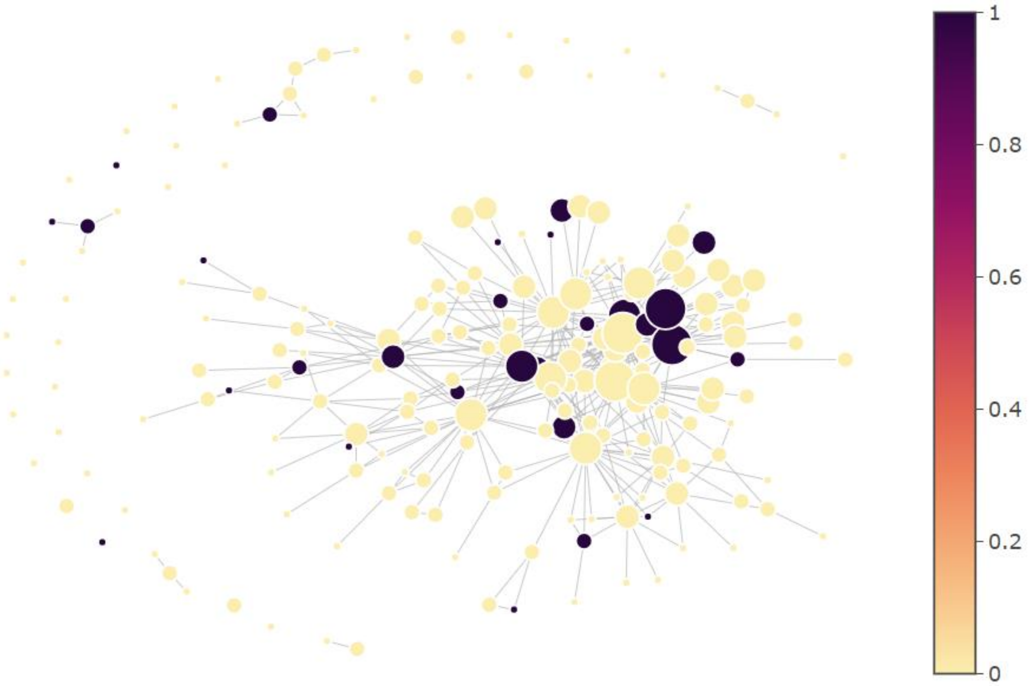

Figure S13. Topological data analysis of drugs in terms of drug classes. The nodes are clusters of drugs and the color indicates the distribution of drug classes. **a** Drug clustering vs. distribution of drugs in Benzenoids class (149 drugs included). **b** Drug clustering vs. distribution of drugs in Nucleosides, nucleotides, and analogues class (9 drugs included). **c** Drug clustering vs. distribution of drugs belonging to AHFS code 28:00:00 (136 drugs included).

d Drug clustering vs. distribution of drugs belonging to AHFS code 24:00:00 (72 drugs included).
