# Supplementary material for: Predicting associations among drugs, targets and diseases by tensor decomposition for drug repositioning
Source: BMC Bioinformatics. 2019 Dec 16;20(Suppl 26):628. doi: 10.1186/s12859-019-3283-6 (PMC6912989; doi:10.1186/s12859-019-3283-6)
Supplement: Supplementary file 14 — Additional file 14 Figure S14. Topological data analysis of diseases. [file 12859_2019_3283_MOESM14_ESM.pdf]

A

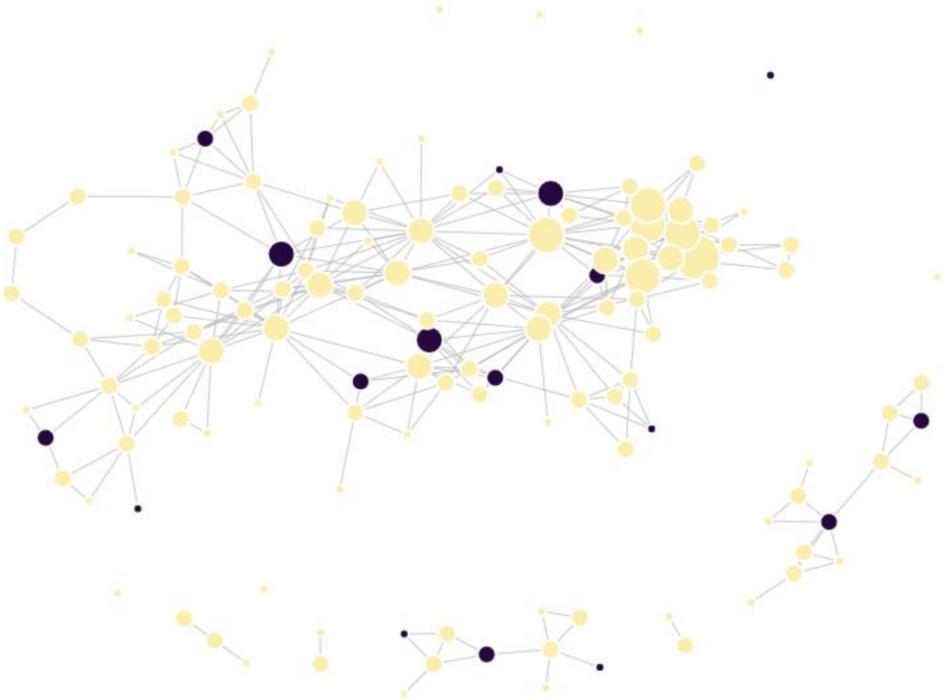

B

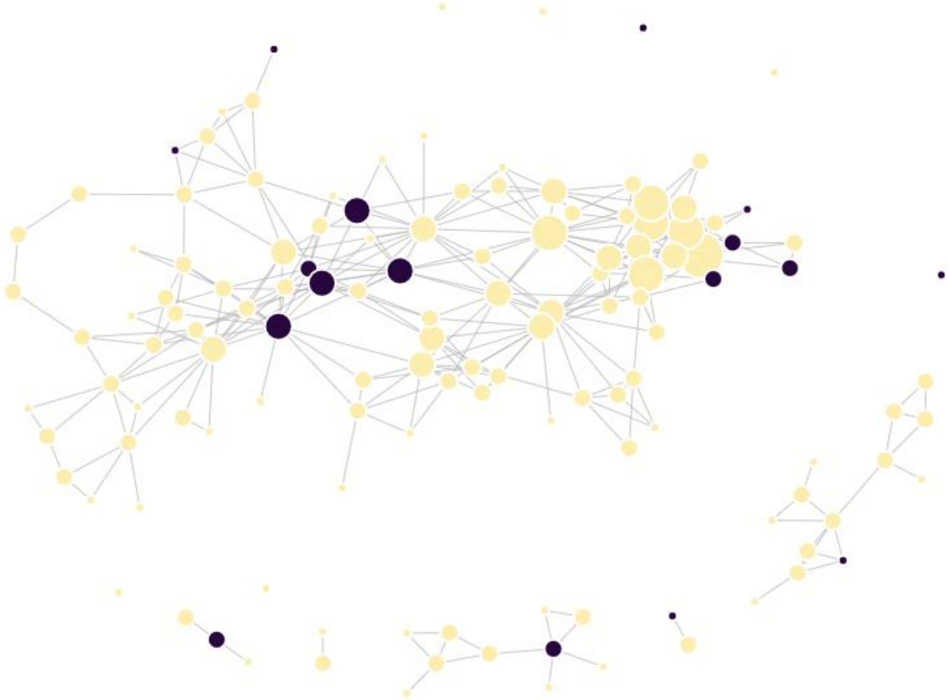

c

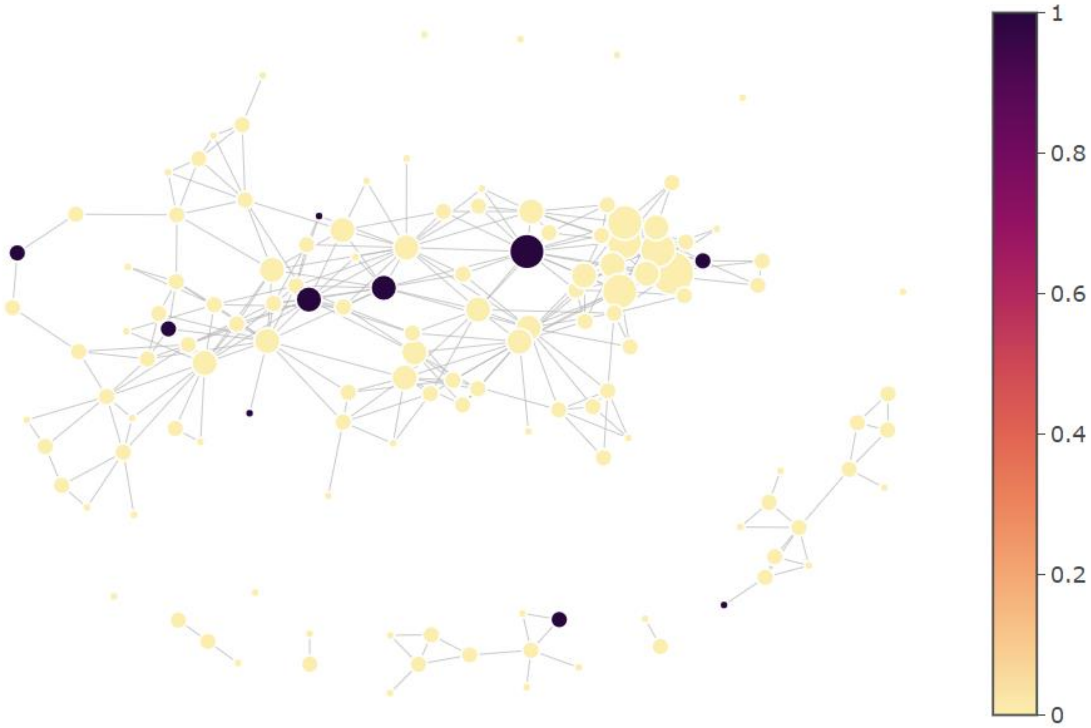

Figure S14. Topological data analysis of diseases. The nodes are clusters of diseases and the color demonstrates the distribution of disease classes. **a** Disease clustering vs. distribution of cancers (48 diseases included). **b** Disease clustering vs. distribution of cardiovascular diseases (38 diseases included). **c** Disease clustering vs. distribution of nervous system diseases (41 diseases included).
