# Supplementary figures and images for: Predicting associations among drugs, targets and diseases by tensor decomposition for drug repositioning
Source: BMC Bioinformatics. 2019 Dec 16;20(Suppl 26):628. doi: 10.1186/s12859-019-3283-6 (PMC6912989; doi:10.1186/s12859-019-3283-6)

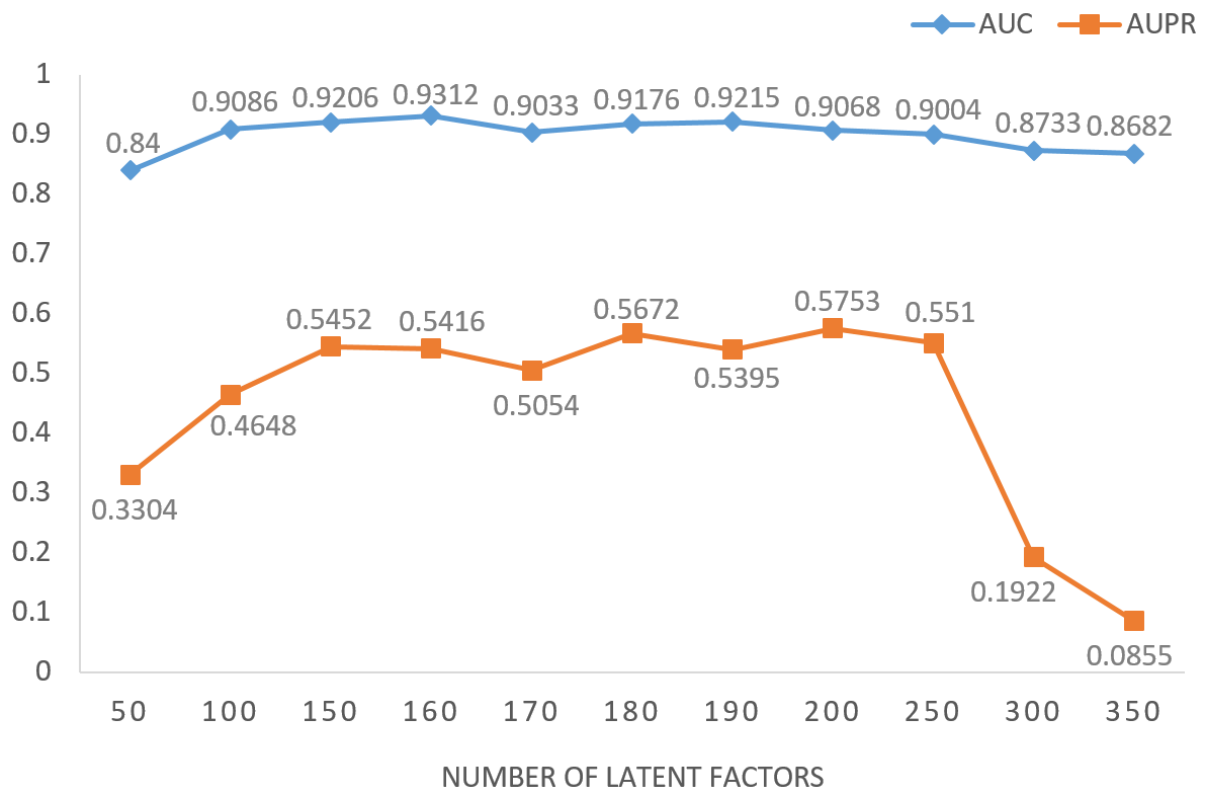

**Figure S15. Performance of CMF with different number of latent factors.**

Supplement: Supplementary file 16 — Additional file 16 Figure S15. Performance of CMF with different number of latent factors. [file 12859_2019_3283_MOESM16_ESM.pdf]
